# The Lysine Acetylation Modification in the Porin Aha1 of *Aeromonas hydrophila* Regulates the Uptake of Multidrug Antibiotics

**DOI:** 10.1016/j.mcpro.2022.100248

**Published:** 2022-05-21

**Authors:** Lishan Zhang, Zujie Yao, Huamei Tang, Qingli Song, Huanhuan Song, Jindong Yao, Zhen Li, Xiaofang Xie, Yuexu Lin, Xiangmin Lin

**Affiliations:** 1 Fujian Provincial Key Laboratory of Agroecological Processing and Safety Monitoring (School of Life Sciences, Fujian Agriculture and Forestry University), Fuzhou, China; 2 Key Laboratory of Crop Ecology and Molecular Physiology (Fujian Agriculture and Forestry University), Fujian Province University, Fuzhou, China; 3 Key Laboratory of Marine Biotechnology of Fujian Province, Institute of Oceanology, Fujian Agriculture and Forestry University, Fuzhou, China; 4 National Key Laboratory of Plant Molecular Genetics, CAS Center for Excellence in Molecular Plant Sciences, Institute of Plant Physiology and Ecology, SIBS, Chinese Academy of Sciences, Shanghai, China; 5 Pharmacy Department, Zhangzhou Health Vocational College, Zhangzhou, China

**Keywords:** antibiotic resistance, quantitative acetylome, aha1, porin permeability, *Aeromonas hydrophila*, ACN, acetonitrile, AMP, ampicillin, AMR, antimicrobial resistance, ARG, antimicrobial resistance gene, CAR, carbenicillin, CARD, Comprehensive Antibiotic Research Database, CS, colistin sulfate, ETP, ertapenem, FA, formic acid, Kac, protein lysine acetylation, LFQ, label-free quantitation, MBC, minimal bacterial concentration, MIC, minimum inhibitory concentration, MS, mass spectrum, MSA, multiple sequence alignment, OXY, oxytetracycline, OXY^R^, oxytetracycline resistance, OXY^S^, oxytetracycline sensitive, PB, polymyxin sulfate B, PG, penicillin G, TET, tetracycline

## Abstract

Protein lysine acetylation (Kac) modification plays important roles in diverse physiological functions. However, there is little evidence on the role of Kac modification in bacterial antibiotic resistance. Here, we compared the differential expressions of whole-cell proteins and Kac peptides in oxytetracycline sensitive and oxytetracycline resistance (OXY^R^) strains of *Aeromonas hydrophila* using quantitative proteomics technologies. We observed a porin family protein Aha1 downregulated in the OXY^R^ strain, which may have an important role in the OXY resistance. Interestingly, seven of eight Kac peptides of Aha1 decreased abundance in OXY^R^ as well. Microbiologic assays showed that the K57R, K187R, and K197R Aha1 mutants significantly increased antibiotic resistance to OXY and reduced the intracellular OXY accumulation in OXY stress. Moreover, these Aha1 mutants displayed multidrug resistance features to tetracyclines and β-lactam antibiotics. The 3D model prediction showed that the Kac states of K57, K187, and K197 sites located at the extracellular pore vestibule of Aha1 may be involved in the uptake of specific types of antibiotics. Overall, our results indicate a novel antibiotic resistance mechanism mediated by Kac modification, which may provide a clue for the development of antibiotic therapy strategies.

## Highlights

• Aha1 plays important role on oxytetracycline resistance.
• The deletion of aha1 reduces intracellular oxytetracycline accumulation.
• The Kac status on Aha1 affects oxytetracycline susceptibility.
• The Kac status on Aha1 involve in the regulation of multidrug antibiotics uptake.

## Graphical Abstract

**Figure:**
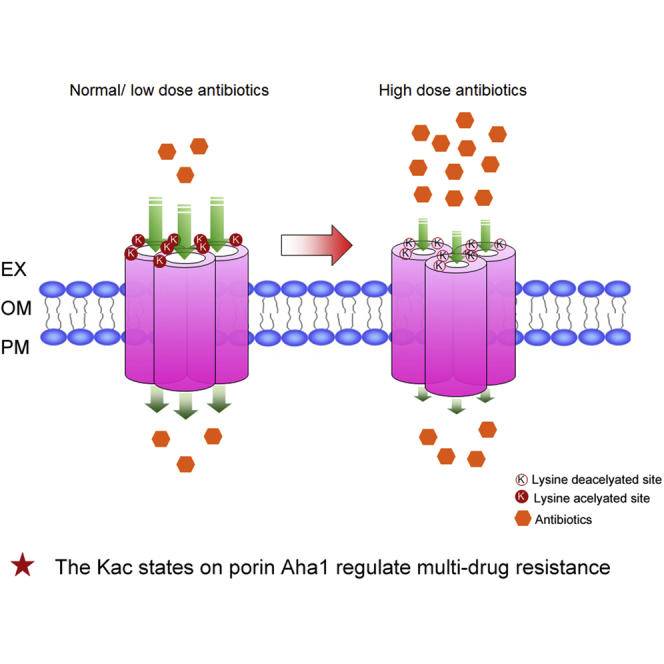

## In Brief

Bacterial antibiotic resistance has become a serious public problem in the world, whereas the intrinsic antibiotic resistance mechanisms are still largely elusive. Protein lysine acetylation (Kac) modification is well distributed in prokaryotic cells and plays important roles in diverse physiological functions. Here, we found that the Kac modifications in a porin family protein Aha1 of *Aeromonas hydrophila* regulate the uptake of multidrug antibiotics, which may provide a clue for the development of antibiotic therapy strategies.

Antibiotics were discovered over 90 years ago, and bacterial antibiotic resistance has become a serious public health problem. Drug-resistant bacterial strains have been found worldwide in environments including clinical hospitals, livestock breeding, and aquaculture. It has been well documented that bacteria have evolved diverse ways to deal with antibiotics, such as pumping intracellular drugs out or using membrane envelope proteins to prohibit the entry of extracellular drugs, alter the drug target structure by gene mutation, and change the intracellular metabolic pathways to adapt to antibiotics attack (1, 2, 3, 4). However, these strategies need to pay energy costs that may be unfavorable for bacteria survival in the complicated environment of bacterial community competition. For intrinsic or adaptive antibiotic resistance, bacteria must quickly respond to the concentration of toxic antibiotics, regulate multiple antimicrobial resistance genes (ARGs), and then trigger the translation machine to express related drug defense proteins. Moreover, except for the antimicrobial resistance (AMR)-related proteins and genes, the expression levels of many other proteins or genes fluctuate for the intracellular homeostasis under antibiotics stress. For example, a total of hundreds of proteins in *Edwardsiella tarda* were altered under oxytetracycline (OXY) stress in our previous work, whereas only a few proteins were confirmed to relate to OXY resistance (5). Furthermore, the homeostasis of intracellular contents needs to consume additional energy to return to a normal state when the environmental antibiotics wear off. However, for the acquired antibiotic resistance, although the mutation or incorporation of ARGs may quickly provide drug resistance during evolution, these gene-deficient or point-mutant strains may experience a fitness cost in the absence of the drug (6). Therefore, quickly obtaining resistance characteristics when responding to antibiotics and then quickly returning to the original state in a nontoxic environment should be an effective strategy for bacteria to survive. However, few of these strategies of bacterial antibiotic resistance have been well characterized so far.

The protein lysine acetylation (Kac) modification is a well-known protein posttranslational modification (PTM), frequent in eukaryotic and prokaryotic cells. Kac modifications in proteins have been reported to play important roles in diverse bacterial biological functions such as metabolism, quorum sensing, chemotaxis, and virulence (7, 8, 9, 10). Because the Kac modification is a reversible and dynamical PTM pattern, whether this modification is involved in bacterial antibiotic resistance has aroused researchers' attention. In fact, some AMR proteins have been identified to have Kac modification sites by combining high-resolution mass spectrometry and Kac immunoaffinity enrichment approaches. For example, the DNA gyrase subunit B, GyrB, that was reported to contribute to bacterial quinolone resistance, has acetylation modifications on several lysine sites in many bacterial species such as *Mycobacterium abscessus* and *Aeromonas hydrophila*, suggesting that GyrB may alter bacterial quinolone resistance *via* Kac modification (8, 11, 12). Furthermore, Li *et al*. reported that the Kac expressions of at least 14 Kac AMR proteins were significantly different between ciprofloxacin-resistant and susceptible strains of *Salmonella typhimurium* (13). However, how bacterial protein regulates antibiotic resistance *via* reversible and dynamical Kac modification, especially directly, is still poorly understood.

To better understand the role of Kac modification in bacterial antibiotic resistance, here, we first compared the differential expression between OXY-sensitive (OXY^S^) and OXY-resistance (OXY^R^) strain of pathogenic *A. hydrophila* in both whole protein and Kac modification peptide levels using quantitative proteomics approaches. We found that an outer membrane protein, Aha1 (gene name *AHA_2785*), was significantly decreased in both whole protein and Kac peptide levels. The functional validation showed that the deletion of *aha1* significantly increased antibiotic resistance against OXY. Moreover, the Kac modifications on this protein's K57, K187, and K197 sites negatively regulated OXY resistance. Finally, we demonstrate that these Kac modifications on Aha1 negatively regulated the entry of OXY into the outer membrane, which was consistent with the OXY resistance data. Moreover, the acetylation modifications in the porin Aha1 of *A. hydrophila* displayed multidrug resistance features. To the best of our knowledge, this is the first report showing that protein Kac modification directly regulates bacterial antibiotic resistance. Our results indicate a novel antibiotic resistance mechanism mediated by PTMs and provide a potential drug target against antibiotic resistance in the future.

## Experimental Procedures

### Chemicals and Reagents

The yeast powder and tryptone were ordered from Bayerdi Biotechnology Co, Ltd. Sodium chloride was obtained from Sinophem Chemical Reagent Co, Ltd. Ligase and 2× Rapid Taq enzyme were obtained from Vazyme Co, Ltd, and the plasmid extraction kit, gel recovery kit, DNA extraction kit, and Mut Express II Fast Mutagenesis Kit were ordered form Magen. All antibiotics were purchased from Aladdin Biotechnology Co, Ltd, including doxycycline, OXY, metacycline, tetracycline (TET), imipenem, aztreonam, ertapenem (ETP), kanamycin, tobramycin, apramycin, gentamicin, erythromycin, lincomycin, roxithromycin, azithromycin, colistin sulfate (CS), polymyxin sulfate B (PB), trimethoprim, pefloxacin, enrofloxacin, ciprofloxacin, enoxacin, levofloxacin, moxifloxacin, carbenicillin (CAR), penicillin G (PG), ampicillin (AMP), latamoxef, cefazolin, cefamandole, and ceftriaxone.

### Bacterial Strains and Growth Conditions

The bacterial strains and plasmids used in this study were *A. hydrophila* ATCC 7966 (OXY^S^) and its antibiotic-induced strain OXY^R^, *Escherichia coli* MC1061-λpir, *E. coli* S17-λpir, plasmids pRE112 and pBBRMCS1. *A. hydrophila* and *E. coli* were cultured in LB medium at 30 °C or 37 °C, respectively. The OXY^R^ strain had a final minimum inhibitory concentration (MIC) to OXY 16-fold higher than the MIC of the original OXY^S^ strain. It was induced from *A. hydrophila* ATCC 7966 by sequential subcultures method as previously described (14). Except for *ΔAHA_0461*, *ΔAHA_1969*, *Δaha1*, *Δomp5*, and *ΔAHA_4275*, mutants that were previously constructed and kept in our laboratory (15), all other mutants and point-mutant derivatives were constructed in this study.

### Whole-Cell Protein Extraction and Digestion

After an overnight incubation for 16 h, the *A. hydrophila* strains were transferred to 30 ml LB medium at 1:100 ratio and cultured in a 30 °C shaker until *A*_600_ = 1.0 and then centrifuged at 5000*g* for 10 min. After washing twice with PBS buffer, 10 ml UA lysate (8 M Urea in 100 mM Tris–HCl pH8.5) was added. The cells were broken with ultrasonication (10 repetitions of the cycle 100 W ultrasonication for 10 s and 10 s on ice), centrifuged at 15,000*g* for 20 min, the supernatant was taken into a new tube, and the protein concentration was determined by Bradford method.

For SDS-PAGE separation, about 20 μg protein samples were taken from each group and added to 5X Laemmli loading buffer, followed by boiling water bath for 5 min and centrifugation at 140,00*g* for 10 min to isolate the supernatant. After a 12% SDS-PAGE electrophoresis, the gel stained with Coomassie bright blue, for whole-cell and Kac modified protein digestion, a total of 12 mg of each sample was added to the final concentration of 10 mM DTT at 37 °C for 2.5 h. Next, we added the final concentration of 50 mM iodoacetamide to the samples and kept them in the dark for 30 min. Then, the concentration of UA buffer was diluted to 1.5 M using five times the volume of water, and trypsin (Promega) was added at a ratio of 1:50 to digest at 37 °C for 18 h. Finally, the digested peptides were desalted by SPE C18 column (Waters WAT051910) and lyophilized by CentriVap concentrator (Labconco Inc) for future mass spectrometry analysis (16).

### Enrichment of Kac Peptides

After lyophilization, 1.4 ml of precooled immunoaffinity purification buffer (50 mM (3-(N-morpholino) propane sulfonic acid), pH 7.2, 10 mM sodium phosphate, and 50 mM NaCl) was added to the peptide samples for redissolution. Pretreated anti-Kac-antibody beads (PTMScan Acetyl-Lysine Motif (Ac-K) Kit, Cell Signal Technology) were added to the samples. The sample was incubated at 4 °C for 1.5 h and then centrifuged at 2000*g* for 30 s. The supernatant was discarded. Anti-Kac antibody beads were cleaned three times with 1 ml precooled immunoaffinity purification buffer and then cleaned three times with 1 ml precooled water. After cleaning, the anti-Kac antibody beads were added to 40 μl 0.15% TFA, kept at room temperature for 10 min, and the procedure was repeated. After centrifugation at 2000*g* for 30 s, the supernatant of the samples was taken and desalted by C18 STAGE Tips (17).

### Label-Free–Based Quantitative Proteomics

For whole-cell proteomics, the desalted peptides were fractionated to six fractions for each sample using a HPLC system (Thermo DINOEX Ultimate 3000 BioRS) and a Welch C18 (5 μm, 120 Å, 4.6 × 250 mm). The peptides samples were dried by vacuum centrifuging and analyzed using TripleTOF 5600 plus coupled with the Eksigent micro LC system (SCIEX). Briefly, peptides were separated by a binary mobile phase system of buffer A (2% ACN (acetonitrile)/0.1% FA (formic acid) in H_2_O) and buffer B (98% ACN/0.1% FA in H_2_O) in a 30 min solvent gradient listed as follows: 5% B, 0 min; 5%–24% B, 20 min; 24%–35% B,5 min; 35%–80% B, 1 min; 80% B, 2 min; 80%–5%,1 min; 5%,1 min. An electrospray voltage of 2.2 kV was applied. The mass spectrometer was programmed to acquire by data-dependent acquisition tandem mass spectra from the top 25 ions in the full scan from 350 to 1500 m/z. Dynamic exclusion was set to 10 s, singly-charged ions were excluded, full mass spectrum (MS) resolution to 30,000 and MS/MS resolution to 15,000. The normalized collision energy was set to 28, automatic gain control to 2e5, max fill of MS to 250 ms, max fill MS/MS to 100 ms.

For Kac-enriched proteomics, the enriched lysine-acetylated peptides were analyzed by LC-MS/MS. HPLC easy-NLC1000 was used for separation with buffer A (2% ACN/0.1% FA in H_2_O) and buffer B (84% ACN/0.1% FA in H_2_O). Chromatographic column Thermo EASY column SC200 150 μm∗100 mm (RP-C18) balanced with 100% liquid A. Samples were fed to Thermo EASY column SC001 traps 150 μm∗20 mm (RP-C18) (Thermo) by the automatic sampler, separated by chromatographic column at a flow rate of 300 nl/min. The relative liquid gradient is as follows: 0 to 110 min, a linear gradient of liquid B ranges from 0% to 55%; 110 to 118 min, a linear gradient of liquid B from 55% to 100%; 118 to 120 min, liquid B maintained at 100%. The hydrolysates were separated by capillary HPLC and analyzed by Q-Exactive mass spectrometry (Thermo Finnigan). Detection method: positive ion, mother ion scanning range: 350 to 1800 m/z, the mass charge ratio of peptide and peptide fragments was collected according to the following methods: 20 fragment maps (MS2 Scan, HCD) were collected after each full scan. MS1 has a resolution of 70,000 at M/Z 200, and MS2 has a resolution of 17,500 at M/Z 200.

All LC-MS/MS original files were searched by Maxquant software (version 1.6.17.0) for identification and quantification against *A. hydrophila* ATCC 7966 database containing 4131 sequences (downloaded from the Uniprot database on 12/23/2019). The common parameter set were as follows: digested by trypsin (specific enzyme digestion of K and R) with two maximum missed cleavage, oxidation (methionine) and acetyl (protein N-term) as the variable modifications, carbamidomethylation as the fixed modification, and the peptide and protein false discovery rate cut-off were set to 0.01. The mass error was set to 20 ppm for the first search, 20 ppm for the main search, and 0.5 Da for fragment ions. For whole-cell proteins, at least two unique peptide matches were required for each protein. For Kac peptide identification, Acetyl(K) as the additional variable modification, max at seven charges, and five modifications per peptide, the best Maxquant Kac peptide score >35 and localization probability >0.75 were used.

### Experimental Design and Statistical Rationale

The whole cell and Kac quantitative proteomics between OXY^R^ and OXY^S^ strain were both performed in three biological replicates. Each biological sample underwent LC-MS/MS analyses. All raw files were imported into MaxQuant software for database search and data analysis. If the label-free quantitation (LFQ) intensity of identified protein was zero in any of the samples, the protein was excluded from further quantification. The protein ratio between OXY^R^ *versus* OXY^S^ >1.5 or <0.667 with *t* test *p*-value<0.05 was considered to be an altered. Besides these parameters, we optimized the Kac peptide identification and quantification criteria for better calculation as follows: (1) If the peptide which has two or three LFQ intensity was zero in the three biological runs of each group, the zero was replaced by the smallest digit except for zero; (2) If only one of the LFQ intensity of identified peptide was zero in the three biological runs of each group, the peptide was excluded from further quantification; (3) If a total of six LFQ intensity were zero in both groups, the peptide was excluded as well. For the peptides that met these criteria, the average LFQ intensity ratio (OXY^R^ *versus* OXY^S^) in three runs >1.5 or <0.667 with *t* test p-value<0.05 was used as a significant change cut-off.

### Bioinformatics Analysis

The altered proteins or Kac peptides found by Kyoto encyclopedia of genes and genomes to be enriched in the whole-cell and Kac proteomics were analyzed by DAVID (https://david.ncifcrf.gov/) (18). The keywords enrichment was performed using the online ShinyGO v0.66 version (http://bioinformatics.sdstate.edu/go/#), and the top 15 terms with the lowest *p*-value were visualized (19). The formula of the up-down normalization score is as follows: $\frac{\text{ Upregulated numbers}-Down\;regulated\;numbers}{Altered\;numbers}$. The circle heatmap was performed by TBtools (20). Amino acid sequence motifs comprising at least 16 amino acids within ±8 residues of the acetylated lysine sites were analyzed using the web-based pLogo software (https://plogo.uconn.edu/#) (21). The database protein sequences of *A. hydrophila* ATCC 7966 from Uniport were used as the background database parameter, whereas the other parameters used the default setting. The amino acid sequences of target proteins were submitted to search against the Comprehensive Antibiotic Research Database (CARD) to predict the AMR protein (22). SWISS-MODEL performed a prediction of 3D protein structure, a protein homology-modeling server with 3D of OmpF in *E. coli* (PDB ID: 6wtz) as the template, and then visualized and analyzed by PyMOL with PyTMs 1.2 and Caver 3.0.3 plugins (23, 24, 25). The multiple sequence alignment (MSA) was analyzed by MEGA v.7.0.26 version and displayed using ESPript 3.0 with slight modifications (26, 27).

### Expression and Purification of the Recombinant Proteins

The recombinant expression protein was constructed as previously described (28). Briefly, the *cobB*, *acuC*, and ah*a1* genes were amplified using *A. hydrophila* ATCC 7966 and as templates respectively using primer pairs listed in supplemental Table S1, and then the target fragments were ligated with the cloning vector pET28a (or pET32a) and transformed into *E. coli* BL21. The positive recombinant strains were inoculated in 150 ml liquid LB, cultured at 37 °C until A600 nm = 0.6, and then incubated at 16 °C for 8 h under 0.6 mM IPTG induction. The cells were collected by centrifugation and washed twice with 1× PBS buffer solution (pH = 7.4) before ultrasonic crushed in binding buffer solution (5 mM imidazole in PBS). The supernatant was gone through a Ni-TNA resin column, washed with 1× PBS buffer, and then eluted with 300 mM imidazole to purify recombinant proteins.

### The Construction of Rescued and Point Mutants

The complementary and site-directed mutagenesis of the target gene was performed as previously described (17). Briefly, a 333 bp DNA fragment upstream of the *aha1* gene was amplified from *A. hydrophila* genomic DNA. A DNA fragment with a 6×His tag sequence was then introduced into the PBBR1-MCS1 plasmid (p*aha1*) at *Hin*dIII and *Bam*HI restriction sites and transformed into competent *E. coli* DH5α cells. Next, PCR and sequencing were performed to verify the correctness of the recovery plasmid. Site-directed mutagenesis was performed using Mut Express II Fast Mutagenesis Kit V2 according to the manufacturer's protocol with the corresponding primers listed in supplemental Table S1. The product was transferred into DNA methylation chemically competent cells. After culturing, the monoclonal clones were selected for bacterial liquid PCR verification with primers *aha1*-P5 and P6 and further verification by sequencing. Finally, the plasmids carrying *aha1*, point mutation *aha1*, and empty vector pBBR1MCS1 were transferred into Δ*aha1* competent cells by electrotransformation to construct rescued and point mutations.

### Western Blotting

Approximately, 20 μg protein samples from each group were separated by 12% SDS-PAGE. Bio-Rad Trans-Blot Semi-Dry Transfer instrument (Procedure: 25V, 15 min) was used to transfer the protein samples in the protein glue to PVDF membrane, and then PBST (8 mM Na_2_HPO_4,_ 0.136 M NaCl, 2 mM KH_2_PO_4,_ 2.6 mM KCl, 0.05%(V/V) Tween-20) was used to wash the membrane for 5 min. After that, a blocking solution (5% skim milk to PBST) was added and the membrane was incubated at room temperature for 1 h. Then anti-Kac antibody (PTM BioLab) was used as the primary antibody at 1:1000 and incubated at 4 °C overnight. The next day, the PVDF membrane was washed with PBST for three times at room temperature, and then the secondary antibody was added at 1: 4000. After incubation at room temperature for 1 h, the membrane was washed with PBST buffer five times. Finally, an ECL chemiluminescence reagent (Bio-Rad) was used for exposure and color rendering in the imager.

### Antibiotics Susceptibility Assay

As previously described, this study used agar dilution to determine the minimal bacterial concentrations (MBCs) to antibiotics (15). Briefly, the bacterial strains (cultured in fresh LB medium until A _600nm_ reached 1.0) were diluted 100 times, and then 2 μl of each sample was spotted on the agar plate containing different concentrations of antibiotics and cultured at 30 °C for 16 h. Each experiment was repeated at least two independent times.

### OXY Accumulation Assay

The OXY accumulation assay was measured by fluorescence spectrometry as previously described (29). The overnight cultured bacterial strains were diluted to fresh LB medium at 1:100 and then incubated to logarithmic growth phase (A _600nm_ = 1.0). The bacterial cells were collected by centrifugation and then suspended in Mg^2+^ buffer (containing 50% methanol,10 mM Tris–HCl, pH 8, 0.1 mM MgCl_2_ and 0.2% glucose). OXY in Mg^2+^ buffer was added to the final concentration of 2 mM and the samples were left standing for 2 h. After centrifugation, the supernatant was removed. The pellets were resuspended in Mg^2+^ buffer and then aliquoted into a 96-well Costar black plate (Corning) at 200 μl) with three well duplicates for each well. The absolute amount of OXY accumulation was measured by a fluorescence spectrophotometer (Molecular Devices) with excitation wavelength at 400 nm and emission wavelength at 520 nm. Each experiment was repeated three independent times.

## Results

### The Differential Expression Between OXY^S^ and OXY^R^*A. hydrophila* Strain in Both Whole-Cell and Kac Modified Samples

**Fig. 1:**
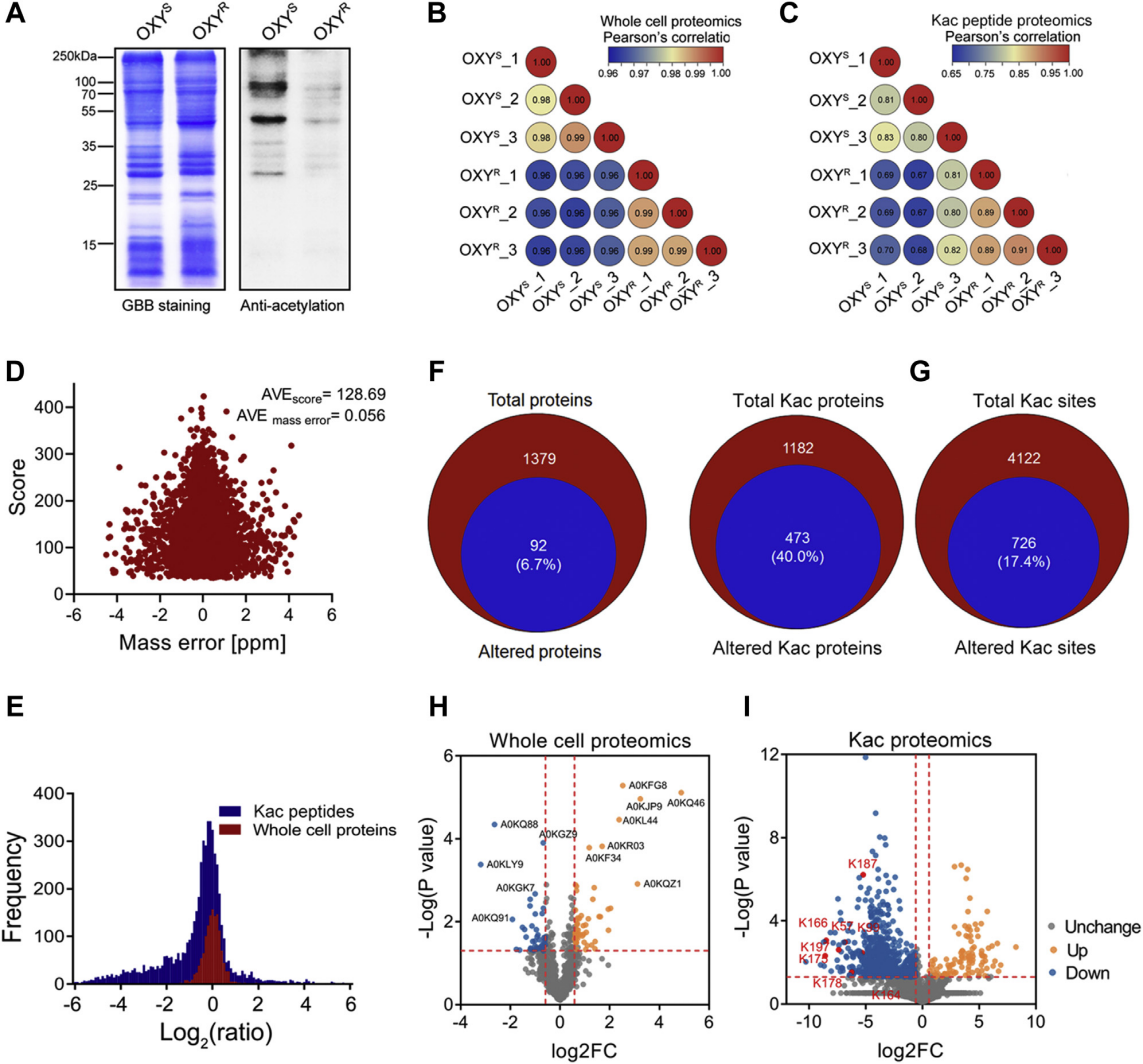
**Whole-cell and Kac peptide proteomics analysis between OXY**^**S**^**and OXY**^**R**^**strains.***A*, the Kac profiles in OXY^S^ and OXY^R^ strains by Western blotting. The *left* figure is the Coomassie blue staining as loading control. *B* and *C*, correlation coefficient analysis of whole-protein and Kac peptide proteomics in three biological repeats for each group, respectively. *D*, the distribution of mass error and the identified score of Kac-modified peptides. *E*, the frequency distribution of protein/peptide ratio (OXY^R^*versus* OXY^S^) in both whole-cell protein and Kac-modified peptide proteomics. *F*, the overlapping proteins between altered and total identified proteins in whole-cell proteomics. *G*, the overlapping Kac proteins/peptides between altered and total identified Kac proteins/peptides in Kac proteomics. *H* and *I*, volcano plot of the *p*-values *versus* the log2 protein abundance differences between OXY^S^ and OXY^R^, with proteins or peptides outside the significance lines colored in *orange* or *blue*, respectively. Some representative protein names and Kac sites are highlighted. OXY^S^, oxytetracycline sensitive; OXY^R^, oxytetracycline resistance; Kac, lysine acetylation.

To better understand the role of Kac modification on bacterial antibiotic resistance, we first detected the Kac modification levels between an OXY^S^ *A. hydrophila* strain and its induced OXY^R^ strain by Western blotting, which had a MIC to OXY 16-fold higher than that of OXY^S^. As shown in Figure 1*A*, when compared with OXY^S^, the Kac level of OXY^R^ sharply decreased, indicating the important role of Kac in antibiotic resistance. Next, we compared the different expression patterns between OXY^R^ and OXY^S^ from whole-cell protein and Kac modification peptide samples using label-free–based quantitative proteomics technologies (supplemental Tables S2 and S3). First, the regression coefficient R correlation factors among biological repeats in both groups were greater than 0.8 in whole-cell proteomics, indicating the stable MS quality and repeatability (Fig. 1*B*). The protein ratios of log2(OXY^R^ *versus* OXY^S^) were normally distributed, and the median is close to zero (log2 scale), suggesting that the altered proteins were unbiased at the protein level (Fig. 1*E*). Therefore, in whole-cell proteomics, we identified 1379 proteins, including 92 proteins (account for 6.7%) that were altered between OXY^R^ and OXY^S^ strains (Fig. 1*F*), of which 54 increased and 38 decreased in abundance (Fig. 1*H*).

In Kac peptide-enriched proteomics, the correlation factors in both group repeats was relatively low (R > 0.65), which may be explained by the experimental variations of laborious PTM enrichment procedures (Fig. 1*C*). We then identified a total of 4122 Kac peptides with considerable high-quality MS, with an average score of 128.69 and an average mass error close to zero (0.056) (Fig. 1*D*). Unlike whole-cell proteomics, the distribution of Kac peptide ratios of log2(OXY^R^ *versus* OXY^S^) has a little bias to a negative value, which was consistent with the previously detected sharp decreased of the Kac level in the OXY^R^ sample by Western blotting (Fig. 1*E*). When compared with the OXY^S^ sample, 726 Kac peptides (corresponding to 474 Kac proteins) of a total of 4122 identified Kac peptides (corresponding to 1182 Kac proteins) were altered in the OXY^R^ sample (Fig. 1*G*). For most of them (628), the levels were decreased, while only 98 Kac peptides showed increased abundance (Fig. 1*I*).

### Bioinformatics Analysis of Altered Proteins and Kac Peptides

**Fig. 2:**
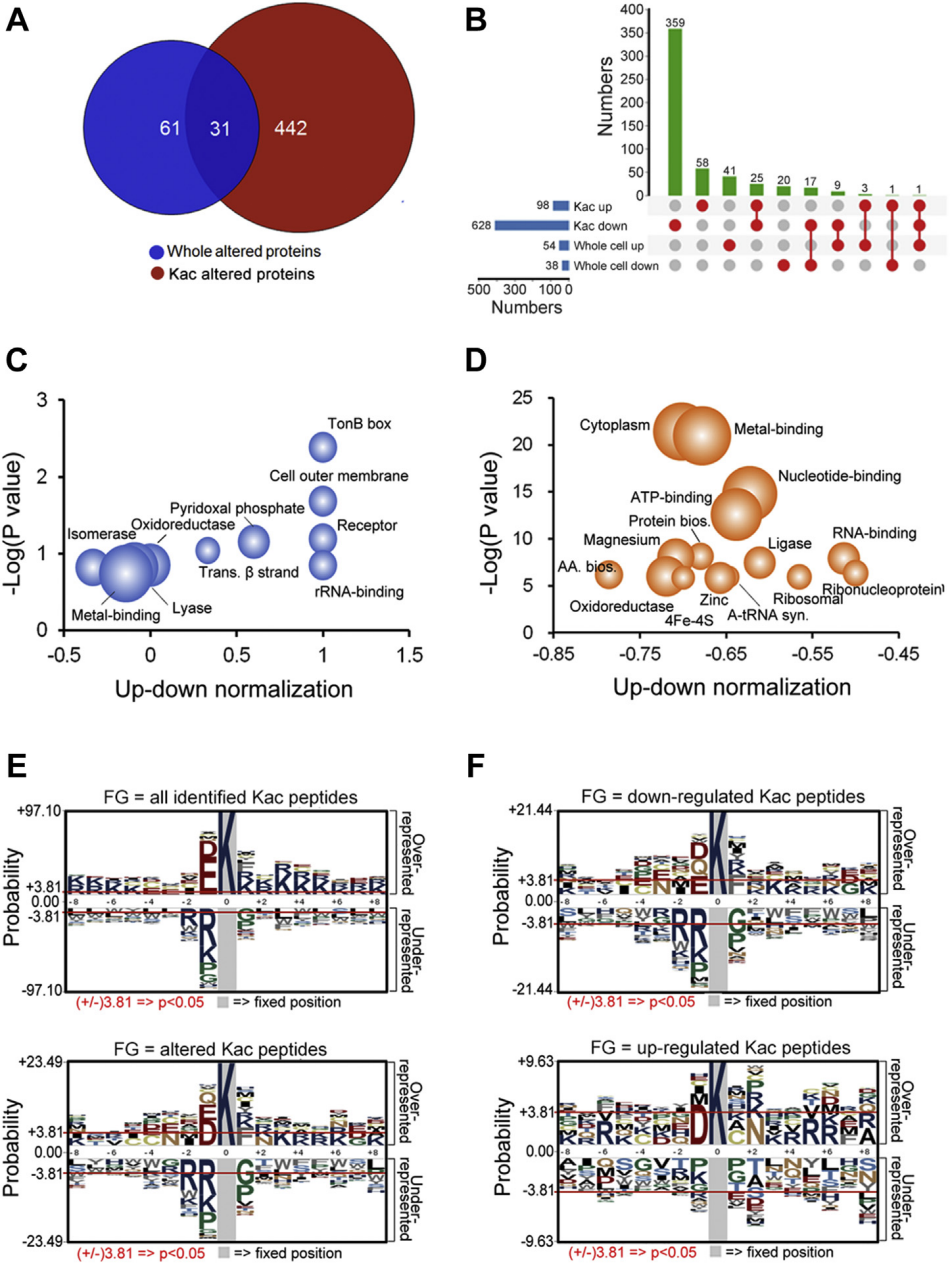
**Bioinformatics and analysis of whole-cell protein and Kac peptide proteomics between OXY**^**S**^**and OXY**^**R**^**strains.***A*, altered proteins comparison between whole-cell and Kac proteomics. *B*, up-set map analysis of differentially expressed proteins between whole-cell and Kac proteomics. *C* and *D*, keyword enrichment of altered proteins with up-down normalization score between whole-cell and Kac proteomics, respectively. The gradient circle size indicates the numbers of altered proteins; *E* and *F*, motif analysis of Kac peptides in *Aeromonas hydrophila*. Sequence models comprising amino acids in specific positions of modified-17-mers (8 amino acids upstream and downstream of the site) of all the protein sequences. The *A. hydrophila* ATCC 7966 protein sequences in the Uniport database are used as the background database parameter. All identified Kac peptides (4122 peptides), altered Kac peptides (726), and upregulated and downregulated Kac peptides (98 and 628) are set as the foreground data. OXY^S^, oxytetracycline sensitive; OXY^R^, oxytetracycline resistance; Kac, lysine acetylation.

We compared the expression patterns between altered whole-cell proteins and Kac proteins. As shown in Figure 2*A*, 31 proteins overlapped between both comparisons, and 17 of them were downregulated, with three proteins (A0KQ46, A0KLY0, and A0KLH4) being both upregulated in whole-cell and Kac-altered proteins (Fig. 2*B*). Interestingly, there was only one protein (SapD) upregulated in Kac proteins and downregulated in whole-cell protein, and one protein (DeoD-2) upregulated and downregulated in different Kac sites and upregulated in whole-cell protein. In addition, 25 Kac proteins that have several Kac sites increased, whereas some others decreased in the OXY^R^ strain, indicating the different biological functions of these Kac sites in the complex physiological processes.

Because the standard gene ontology terms classification failed to find enrichments in the whole-cell proteomics data, we then analyzed the functional category of altered proteins using keywords as enrichment terms with up-down normalization scores to indicate the tendentiousness of altered proteins. In whole-cell altered proteins, upregulated proteins were enriched in TonB box, receptor, outer cell membrane, pyridoxal phosphate, transmembrane beta-strand, and rRNA-binding, whereas downregulated proteins preferred isomerase, oxidoreductase, and metal-binding (Fig. 2*C* and supplemental Table S4). In Kac-altered proteins, all enriched proteins were downregulated proteins. The top five terms with the lowest *p*-value were cytoplasm, metal-binding, nucleotide-binding, ATP-binding, and protein biosynthesis (Fig. 2*D* and supplemental Table S5). These results suggest differences in the enriched cluster characteristics between bacterial antibiotic resistance–related whole-cell and Kac protein levels.

We further investigated the motif characteristics of Kac proteins. As showed in Figure 2*E* and supplemental Table S6, all 4122 identified lysine sites could be enriched (*p* < 00.5) in many motifs, such as EK, DK, KxxK, KxxxK, and KxxxxK (x represents any amino acid residue). In contrast, altered Kac sites have only four significant motifs: DK, EK, EK, KF, and KxxK. This observation indicates that drug-resistant–related sites may prefer these motifs to the other nonspecific Kac sites. Moreover, the DK motif is more frequent in the upregulated Kac sites, whereas the KF, EK, QK, DK, and NxxK motifs are more frequent in the downregulated Kac sites Figure 2*F* and supplemental Table S6.

**Fig. 3:**
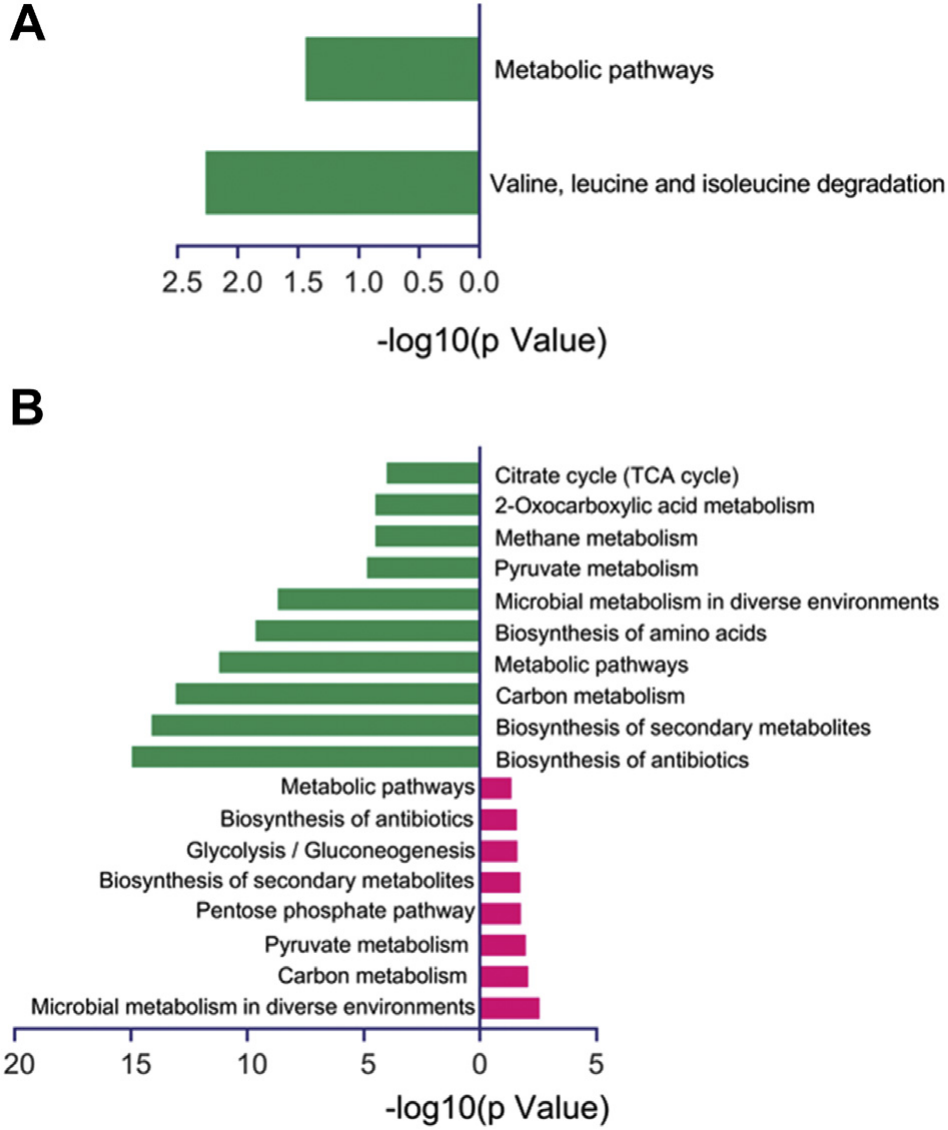
**KEGG enrichment analysis of differential proteins in the whole-cell and Kac proteomics.** (A) and (B) display differentially expressed protein KEGG pathways in the whole-cell and Kac proteomics, respectively. The *green bar* represents KEGG pathways enriched in downregulated proteins/Kac protein ns. The *red bar* is in upregulated proteins/Kac proteins. KEGG, Kyoto encyclopedia of genes and genomes; Kac, lysine acetylation.

Moreover, Kyoto encyclopedia of genes and genomes enrichment analysis was conducted for these differentially expressed proteins. In Figure 3*A*, the downregulated proteins are mainly related to valine, leucine, and isoleucine degradation pathways, whereas the upregulated proteins show no enrichment. For Kac proteomics, despite the significant differences between upregulated and downregulated Kac protein numbers, both were involved in several central metabolic pathways, such as carbon metabolism, pyruvate metabolism, and biosynthesis of antibiotics (Fig. 3*B* and supplemental Table S7). That was consistent with the previous conclusions that Kac modifications are widely distributed in the enzymes in central metabolism pathways in eukaryotic and prokaryotic cells (30).

### The Deletion of *aha1* Increased Bacterial Antibiotic Resistance to OXY

**Fig. 4:**
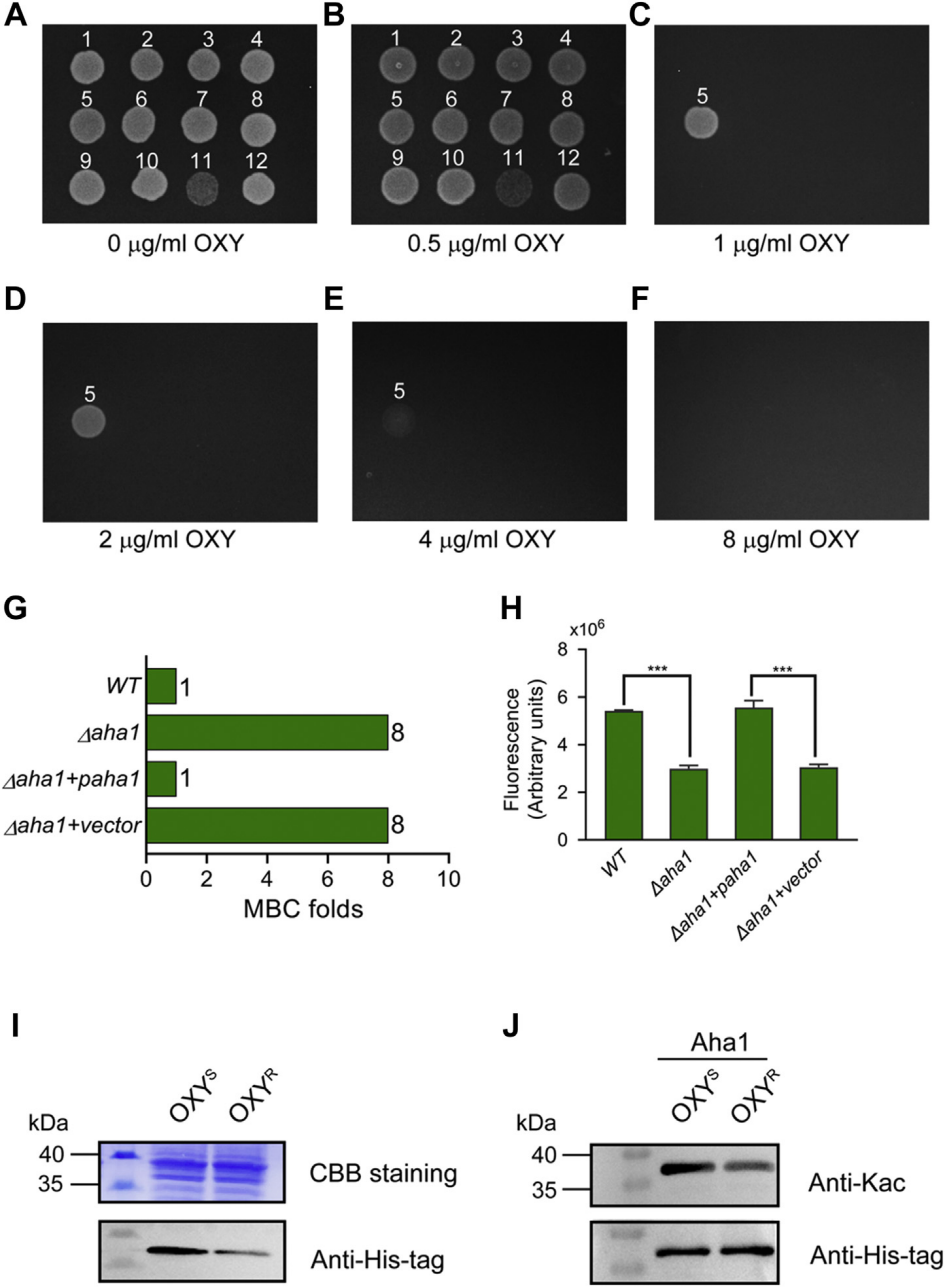
**Aha1 in *Aeromonas hydrophila* plays an important role in the OXY resistance.***A*–*F*, the antibiotic susceptibility assay of 11 *Aeromonas hydrophila* gene deleted mutants to oxytetracycline (OXY) using the MBC plate method. The knockout genes are numbered for convenience in the following order: 1, WT; 2, Δ*AHA_0416*; 3, Δ*AHA_1969*; 4, Δ*oppD*; 5, Δ*aha1*; 6, Δ*fdoG*; 7, Δ*omP5*; 8, Δ*fepA*; 9, Δ*AHA_4275*; 10, Δ*fklB*; 11, Δ*iscS*; 12. Δ*sapD*. *G*, histogram displays MBCs of *Δaha1*, rescued *aha1*, WT, and *Δaha1* carrying the empty vector against OXY. *H*, determination of intracellular OXY accumulation of *aha1* deficient and rescued mutants under 2 mM OXY for 2 h. *I*, Aha1 protein expression levels in *aha1* rescued (6xHis tag) OXY^S^ and OXY^R^ strains, respectively. *J*, when the amounts of purified Aha1 proteins were adjusted to the same level, the Kac levels in *aha1* rescued (6xHis tag) OXY^S^ and OXY^R^ strain, respectively. MBC, minimal bacterial concentration; OXY^S^, oxytetracycline sensitive; OXY^R^, oxytetracycline resistance; Kac, lysine acetylation.

We then asked about the role of altered proteins in OXY resistance. A total of 11 genes, including seven genes (*AHA_0461*, *AHA_1969*, *omP5*, *fepA*, *AHA_4275*, *fklB*, *iscS*) encoding proteins that increased, and four genes (*oppD*, *aha1*, *fdoG*, *sapD*) encoding proteins that decreased in the OXY^R^ strain in whole-cell proteomics, were traceless knocked out in *A. hydrophila* ATCC 7966 (supplemental Fig. S1). Their antibiotic susceptibility against OXY was valued by plate MBC assay (Fig. 4, *A*–*F*). When compared with the WT strain, the deletion of *aha1* significantly increased the MBC of OXY eight times, while other mutants did not show significant drug resistance to OXY, suggesting that *aha1* may play an important role in the resistance to OXY antibiotics in *A. hydrophila*. Furthermore, the *aha1* rescued strain restored the OXY susceptibility phenotype, further validating the role of Aha1 in OXY resistance (Fig. 4*G*).

### The Deletion of *aha1* Decreased Porin Permeability to OXY Accumulation in *A. hydrophila*

According to the protein description in Uniport, the Aha1 (Uniport ID A0KLY9, gene name *AHA_2785*) is an outer membrane protein that belongs to the Gram-negative porin family, we speculated that Aha1 might affect the uptake of OXY into the cells. Therefore, fluorescence spectroscopy was used to detect the intracellular accumulation of OXY among *Δaha1*, *aha1* rescued, and their control strains under a high dose of OXY stress. As shown in Figure 4*H*, the accumulation of intracellular OXY in Δ*aha1* was significantly reduced, whereas its rescued strain restored the OXY concentration to the level of the WT *A. hydrophila*. Therefore, our results confirmed that the outer membrane protein *aha1* might influence bacterial drug resistance through outer membrane permeability.

We then further detected the expression of Aha1 in OXY^S^ and OXY^R^ strains by using Western blotting and found that Aha1 was significantly decreased in OXY^R^ strain. Moreover, the Aha1 protein in both strains was purified, respectively. When the amounts of purified proteins were adjusted to the same level, the Kac level was significantly decreased in OXY^R^ strain (Fig. 4,*I* and *J*). These data further confirmed our whole cell protein and Kac quantitative proteomics results.

### The Kac Modification Sites of Aha1 Affected Bacterial OXY Resistance

**Fig. 5:**
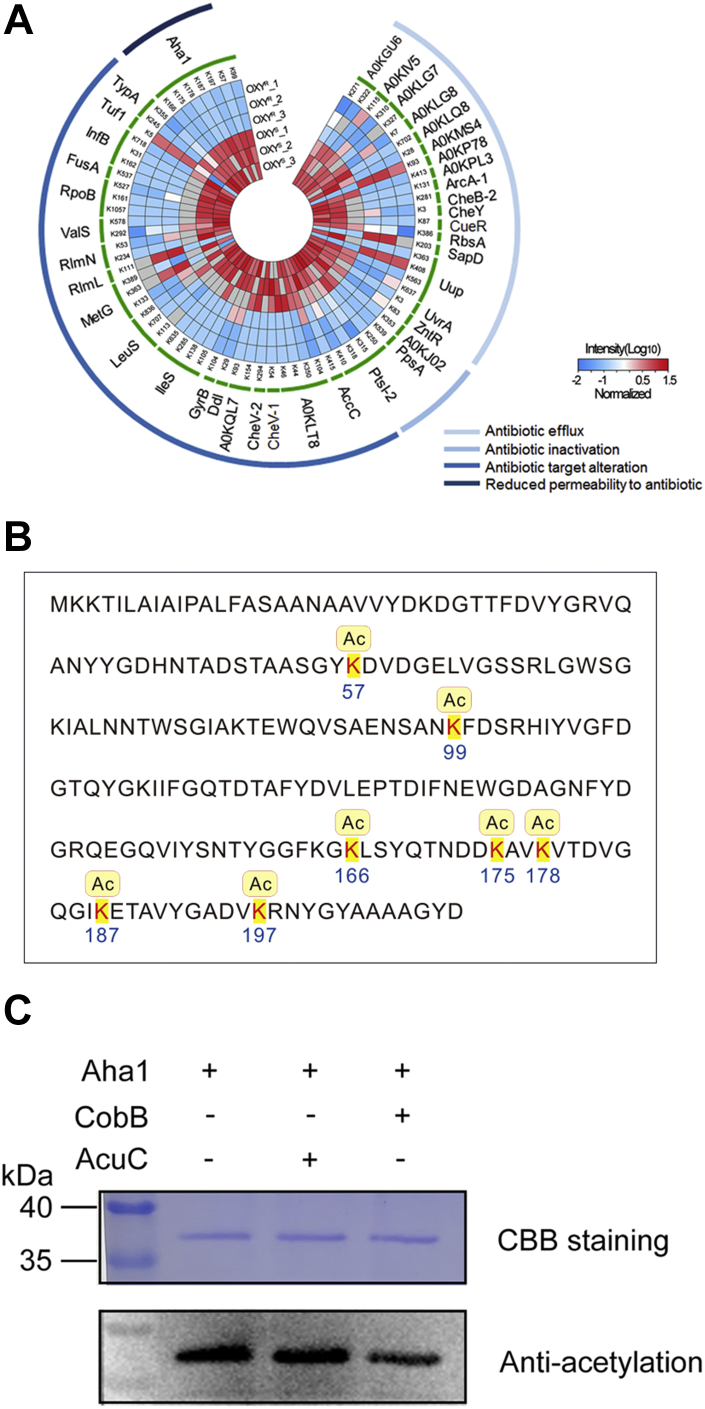
**Lysine acetylation modifications of a total of 71 altered Kac sites in 39 AMR proteins between OXY**^**S**^**and OXY**^**R**^**.***A*, the internal heatmap displays the normalized MS intensity of altered Kac sites in three biological repeats for each group (log10 scale). The corresponding Kac proteins are shown in the *middle*. The *outer circle* displays the four clusters of antibiotic resistance mechanisms according to the CARD database homologous searching. The gradient color indicates the normalized MS intensity (on a log10 scale). *B*, lysine acetylation modifications in the Aha1 sequence of *Aeromonas hydrophila*. *C*, the lysine acetylated Aha1 was deacetylated by deacetylase CobB. OXY^S^, oxytetracycline sensitive; OXY^R^, oxytetracycline resistance; Kac, lysine acetylation; AMR, antimicrobial resistance; MS, mass spectrum; CARD, Comprehensive Antibiotic Research Database.

The potential antibiotic resistance roles of Kac proteins were further analyzed by homologous searching altered Kac proteins in OXY^R^ strain against CARD database, and a total of 39 AMR genes/proteins that have Kac modifications were detected. The Kac sites of these AMR proteins and their MS intensities in Kac proteomics and their possible resistance mechanisms predicted from CARD were displayed in (Fig. 5*A*). According to resistance mechanisms, these proteins could be classified into four classes, antibiotic efflux, antibiotic activation, antibiotic target alteration, and reduced permeability to the antibiotic. Of the 39 AMR Kac proteins, Aha1, PtsI-2, Uup, IleS, and A0KLT8 were the top five abundant Kac sites, while many proteins, such as AccC, UvrA, CheY, CueR, and GyrB, have only one altered Kac site. Most altered Kac sites were downregulated, except for K5 on Tuf1, K111 on RlmL, K363 on MetG, K93 on A0KP78, K203 on SapD, and K408 on Uup. Several Kac sites, K408 and K363 on Uup, K363, and K133 on MetG, for example, displayed opposite modification patterns in one protein, indicating the delicate regulation of Kac modifications on bacterial antibiotic resistance.

The current quantitative acetylome comparison between OXY^S^ and OXY^R^ showed that Aha1 has eight Kac sties (K57, K99, K166, K175, K164, K178, K187, and K197), and seven of them (except for K164) were downregulated in the OXY^R^ stain (Fig. 5*B*). We then asked the lysine acetyltransferase or deacetylase responding for Kac level of Aha1. Firstly, the deletion of *A. hydrophila cobB* (NAD^+^-dependent deacetylase sirtuin family) did affect the bacterial growth in OXY stress, while Δ*acuC* (zinc-dependent deacetylase Rpd3/Hda1 family) and Δ*AHA_0149* (lysine acetyltransferase family) did not, indicating that *cobB* may involve in OXY resistance (supplemental Fig. S2). Moreover, CobB can significantly reduce the Kac level of Aha1 in a manner, whereas AcuC did not. These results further confirmed that CobB is the eraser of Kac Aha1 in *A. hydrophila* (Figs. 5*C* and S3).

**Fig. 6:**
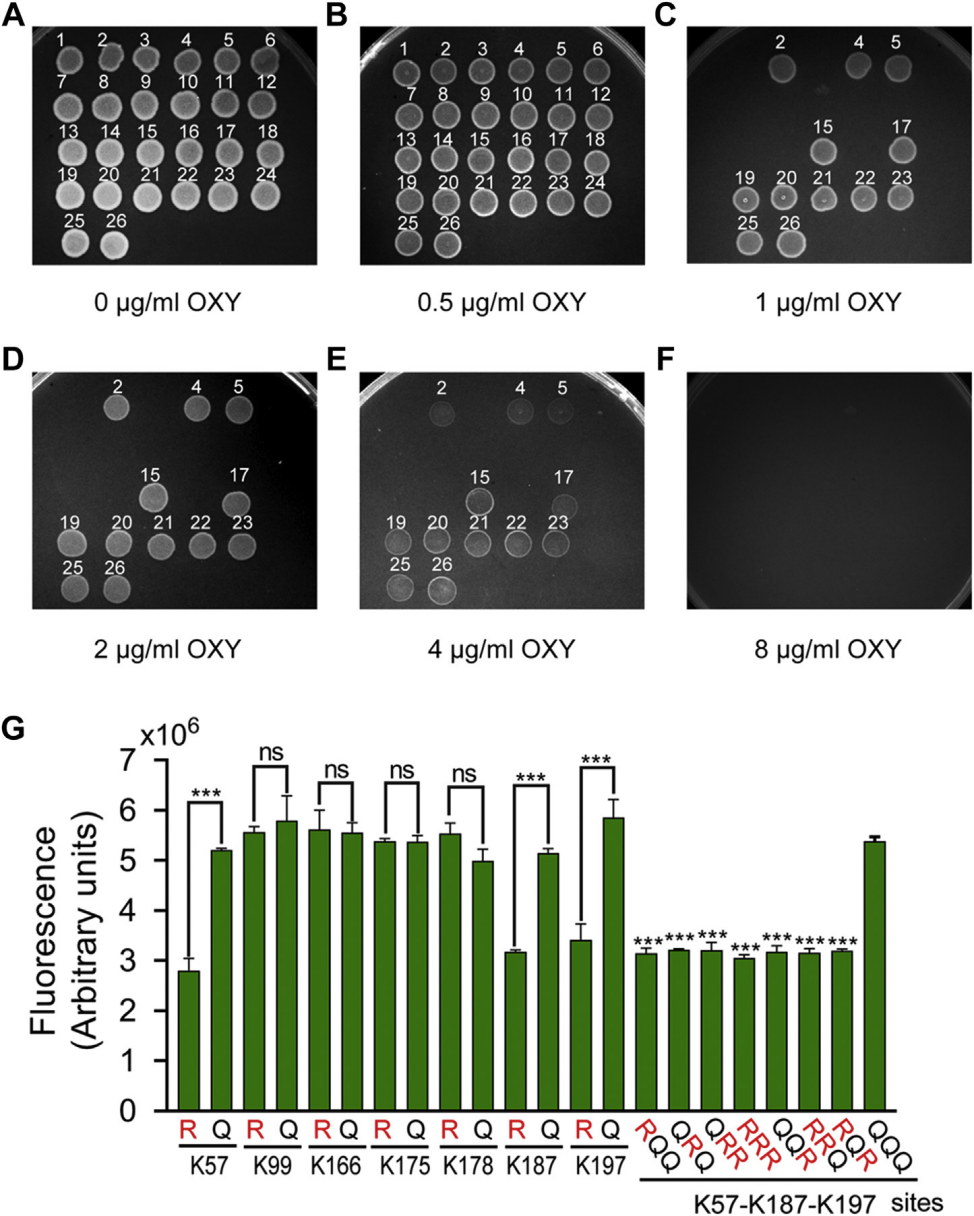
**Several Kac modification sites of Aha1 affect OXY resistance and intracellular OXY accumulation.***A*–*F*, the antibiotic susceptibility assay of *Δaha1* and site-directed mutagenesis generated *aha1* mutants to OXY using the MBC plate method. The *aha1* mutants are numbered in the following order: 1, WT; 2, Δ*aha1*; 3, Δ*aha1*+*paha1*; 4, Δ*aha1*+vector; 5, Δ*aha1*+*paha1-K57R*; 6, Δ*aha1*+*paha1-K57Q*; 7, Δ*aha1*+*paha1-K99R*; 8, Δ*aha1+paha1-K99Q*; 9, Δ*aha1*+*paha1-K166R*; 10, Δ*aha1*+*paha1-K166Q*; 11, Δ*aha1*+*paha1-K175R*; 12, Δ*aha1*+*paha1-K175Q*; 13, Δ*aha1*+*paha1-K178R*; 14, Δ*aha1*+*paha1-K178Q*; 15, Δ*aha1*+*paha1-K187R*; 16, Δ*aha1*+*paha1-K187Q*; 17, Δ*aha1*+*paha1-K197R*; 18, Δ*aha1*+*paha1-K197Q*; 19, Δ*aha1*+*paha1-K57R-K187Q-K197Q*; 20, Δ*aha1*+*paha1-K57Q-K187R-K197Q*; 21, Δ*aha1*+*paha1-K57Q-K187R-K197R*; 22, Δ*aha1*+*paha1-K57R-K187R-K197R*; 23, Δ*aha1*+paha1-*K57Q-K187Q-K197R*; 24, Δ*aha1*+*paha1-K57Q-K187Q-K197Q*; 25, Δ*aha1*+*paha1-K57R-K187R-K197Q*; 26, Δ*aha1*+*paha1-K57R-K187Q-K197R*. *G*, determination of intracellular OXY accumulations in *aha1* mutants under 2 mM OXY for 2 h. OXY, oxytetracycline; Kac, lysine acetylation; MBC, minimal bacterial concentration.

Combined with the clues mentioned above for the phenotypes of Aha1 against OXY stress, we were interested in the effect of Kac modifications of this porin protein on OXY resistance. First, the seven Kac sites were mutated to arginine (R) or glutamine (Q) to mimic lysine deacetylation and acetylation modifications, respectively (supplemental Fig. S4）. Next, the plate MBCs assays of Aha1 derivatives against OXY showed that K57R, K187R, and K197R of Aha1 derivatives significantly increased OXY resistance, and their MBCs to OXY were eight-fold higher than the corresponding K57Q, K187Q, and K197Q derivatives (Fig. 6, *A*–*F*). We then further investigated the relationship among the three Aha1 mutants by multiple site-directed mutagenesis. As shown in Figure 6, when compared with the K57Q-K187Q-K197Q mutant, any sites of K57, K187, or K197 mutated to R all increased antibiotic resistance eight times MBCs to OXY. Most interestingly, the effects of any of Aha1 K57R, K187R, and K197R derivatives on OXY susceptibility equaled to the behaviors of *Δaha1* strain, suggesting that the deacetylations of K57, K187, and K197 play an important role in the OXY resistance and the role of these three Kac sites on OXY resistance should be independent. Moreover, the following OXY accumulation assays were consistent with the antibiotic resistance characteristics of these Aha1 derivatives (Fig. 6*G*). Besides these, we also constructed *aha1* deleted, rescued, and site-specific mutagenesis mutants in an *A. hydrophila* clinical pathogen strain namely LP 2 that was isolated from diseased *European eel* (*Anguilla anguilla*) with a considerable OXY resistance characteristic (supplemental Fig. S5, *A* and *B*). When compared to WT *A. hydrophila* LP-2 strain, the deletion of *lpaha1* significantly increased the MBC of OXY four times. The plate MBCs assays showed that K57R, K187R, and K197R of LpAha1 derivatives significantly increased OXY resistance, and their MBCs to OXY were four-fold higher than the corresponding K57Q, K187Q, and K197Q derivatives (supplemental Fig. S5, *C*–*H*). These findings further confirmed that Aha1 plays an important role in bacterial OXY resistance and provides a novel mechanism that Kac modifications may affect the uptake of OXY into the cell by regulating the Kac modification status on specific lysine sites of this protein.

### The Kac Modification Sites of Aha1 may Affect the Porin Channel for Antibiotics Uptake

**Fig. 7:**
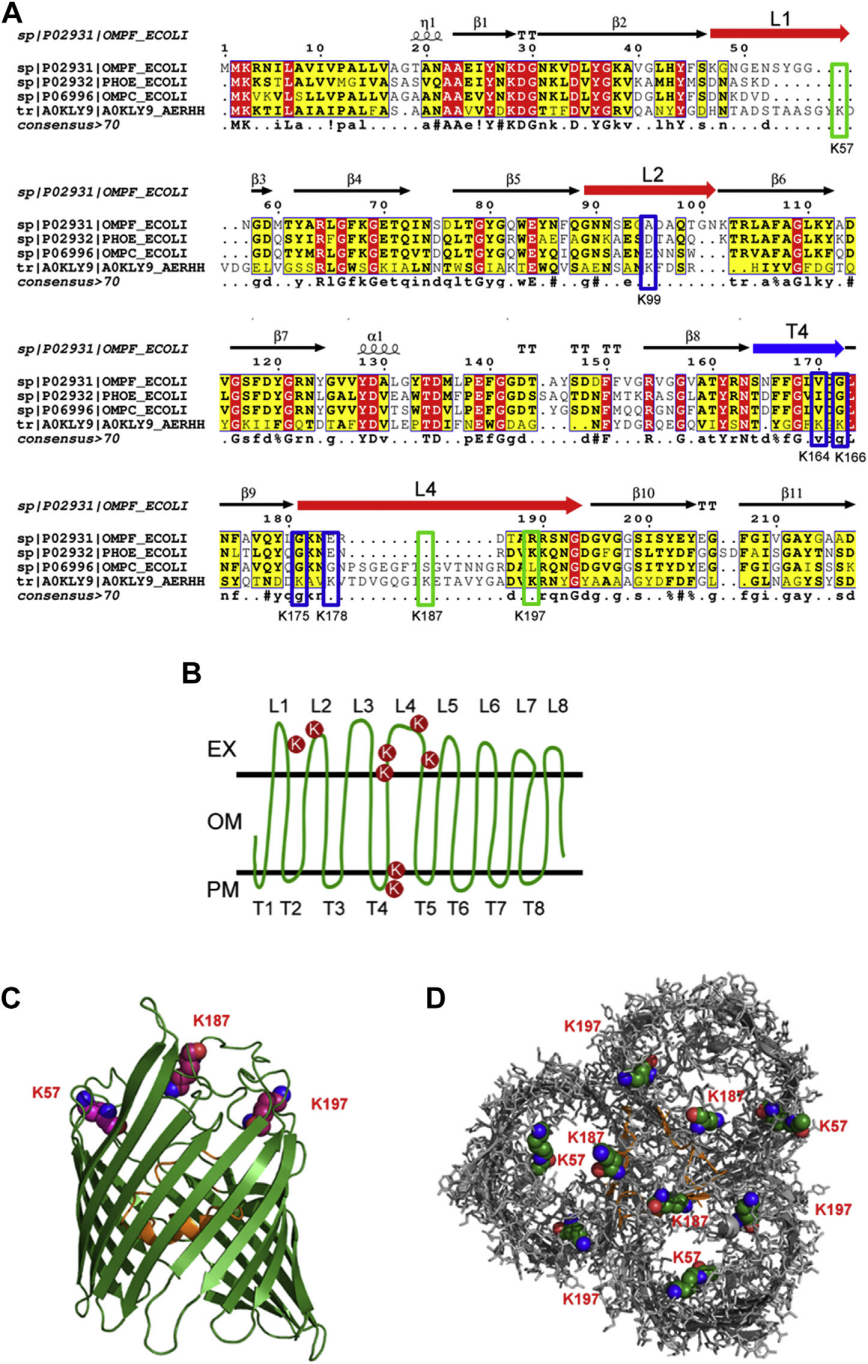
**The 3D structure prediction of lysine acetylation modification Aha1.***A*, structure-based partial MSA of OmpF, OmpC, and PhoE in *Escherichia coli* and Aha1 (A0KLY9) in *Aeromonas hydrophila* ATCC 7966 are presented with ESPript 3.0. The five inactive Kac residues within the *blue* frame and three active Kac residues (K57, K187, and K197) in Aha1 within the *green* frame are highlighted. The ranges of loop1 (L1), loop2 (L2), and loop4 (L4) are presented as a *red arrow*, and turn4 (T4) is presented as a *blue arrow*. *B*, schematic illustrations of the Aha1 with eight Kac sites marked with *red circles*. EX, OM, and PM indicate extracellular, outer membrane, and periplasmic space, respectively. *C*, side view of the monomeric unit of Aha1. The loop3 are visualized in *orange color*. *D*, trimeric porin Aha1 are shown in *top* view. The loop3 are visualized in *orange color*. The atoms of three active residues (K57, K187, and K197) with Kac modifications are shown in a *sphere* representation. MSA, multiple sequence alignment; Kac, lysine acetylation.

**Fig. 8:**
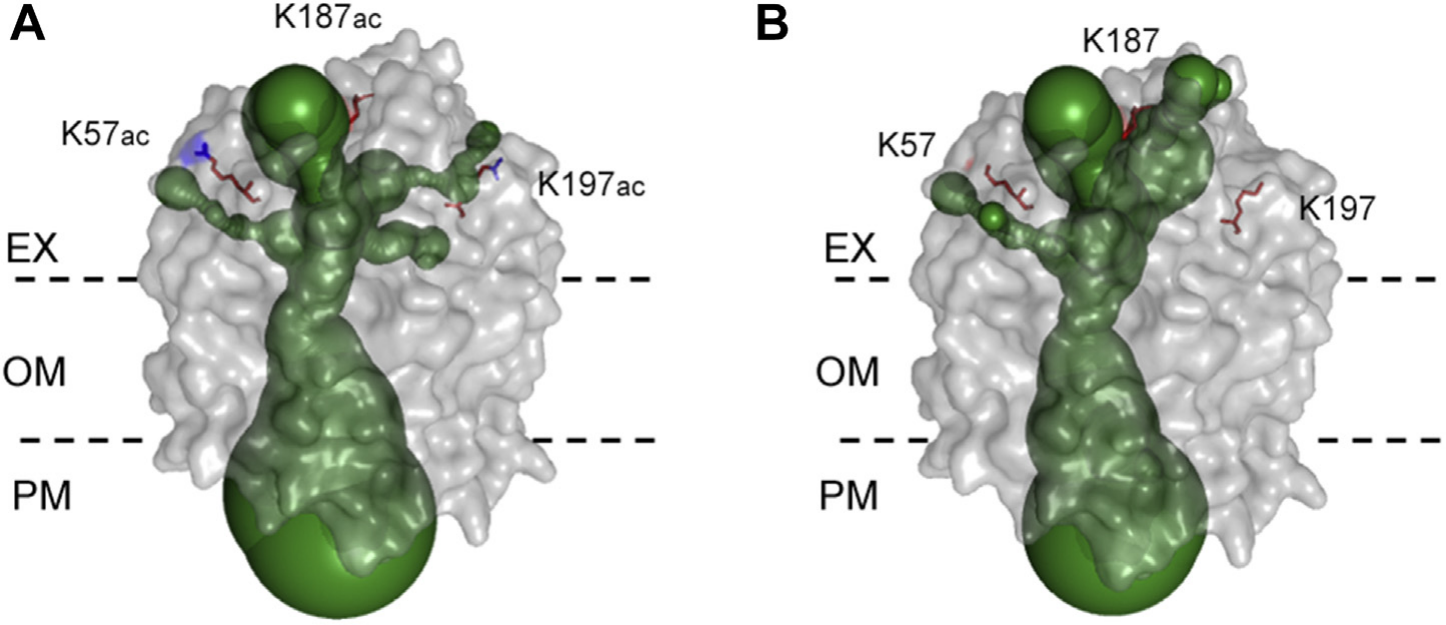
**The channel prediction between lysine acetylation/deacetylation modification Aha1.** 3D structure channels between lysine acetylation (A) and deacetylation modification (B) Aha1 are predicted by Caver 3.0.3, respectively. The predicted tunnel in 3D structure Aha1(in a *grayish* surface representation) is depicted as *green spheres*. The three active residues (K57, K187, and K197) are shown as *red sticks*, and the additional Kac modifications are shown as *blue sticks*. EX, OM, and PM indicate extracellular, outer membrane, and periplasmic space, respectively. Kac, lysine acetylation.

We then asked about the OXY resistance mechanism mediated by Kac sites on *A. hydrophila* Aha1 using MSA and 3D structure prediction. As a member of the porin family, Aha1 shares a considerably high identity with *E. coli* OmpF, PhoE, and OmpC. Those 3D structures as homotrimers were well documented, and their roles as antibiotic uptake channels were well characterized. According to the X-ray crystallographic analysis of 3D structure, *E. coli* OmpF is a homotrimer. The traditional prion monomer has a series of 16 antiparallel β-strands connected by eight external loops (L) and eight periplasmic turns (T) (31). Loop2 plays an important role in stabilizing a trimeric form, and Loop3 narrows the channel acting as a molecular filter (Fig. 7*A*) (32). We then built the 3D modeling prediction of Aha1 using *E. coli* OmpF as a homologous template. Of these eight identified Kac sites, K57 is located in loop1, K99 is in loop2, K164 and K166 are located in turn 4, K175 is in the β9 strand, and K178, K187, and K197 are located in loop4 (Fig. 7*B*). Except for K164, all other Kac sites of Aha1 were downregulated in the OXY resistance strain. Since the important role of Kac/lysine deacetylation modifications on K57, K187, and K197 sites of Aha1 in OXY resistance, the Kac modifications of Aha1 on these three sites were highlighted in its 3D predicted model in monomer and homotrimer (Fig. 7, *C* and *D*). The K57, K187, and K197 sites were all located in the extracellular loops. Moreover, The PyMOL plugin of Caver 3.0.3 was employed to predict the effect of Kac states on the channel of this porin. As showed in Figure 8, the Kac states did not affect the porin size of its intracellular space but significantly change the cavities within extracellular aspect. There were at least three paths directed to the central tunnel. The lysine acetylated K57, K187, and K197 sites of Aha1 were close to these paths and may affect their binding to antibiotics. Although the deacetylation of K57 and K187 sites does not seem to affect the bottlenecks of channel significantly, the different charge characters of both residues (from positive to null charge) may affect the interaction between Aha1 and antibiotics. Most interesting, the deacetylation of K197 site significantly changed the size and direction of the branch of tunnel that prefer to close to Kac site. Therefore, our results indicate that the switch of the acetylation status may affect the pore size of Aha1 porin and then regulate the uptake of OXY antibiotics.

### The Kac States of K57, K187, and K197 sites on Aha1 Mediate *A. hydrophila* Multidrug Resistance

**Fig. 9:**
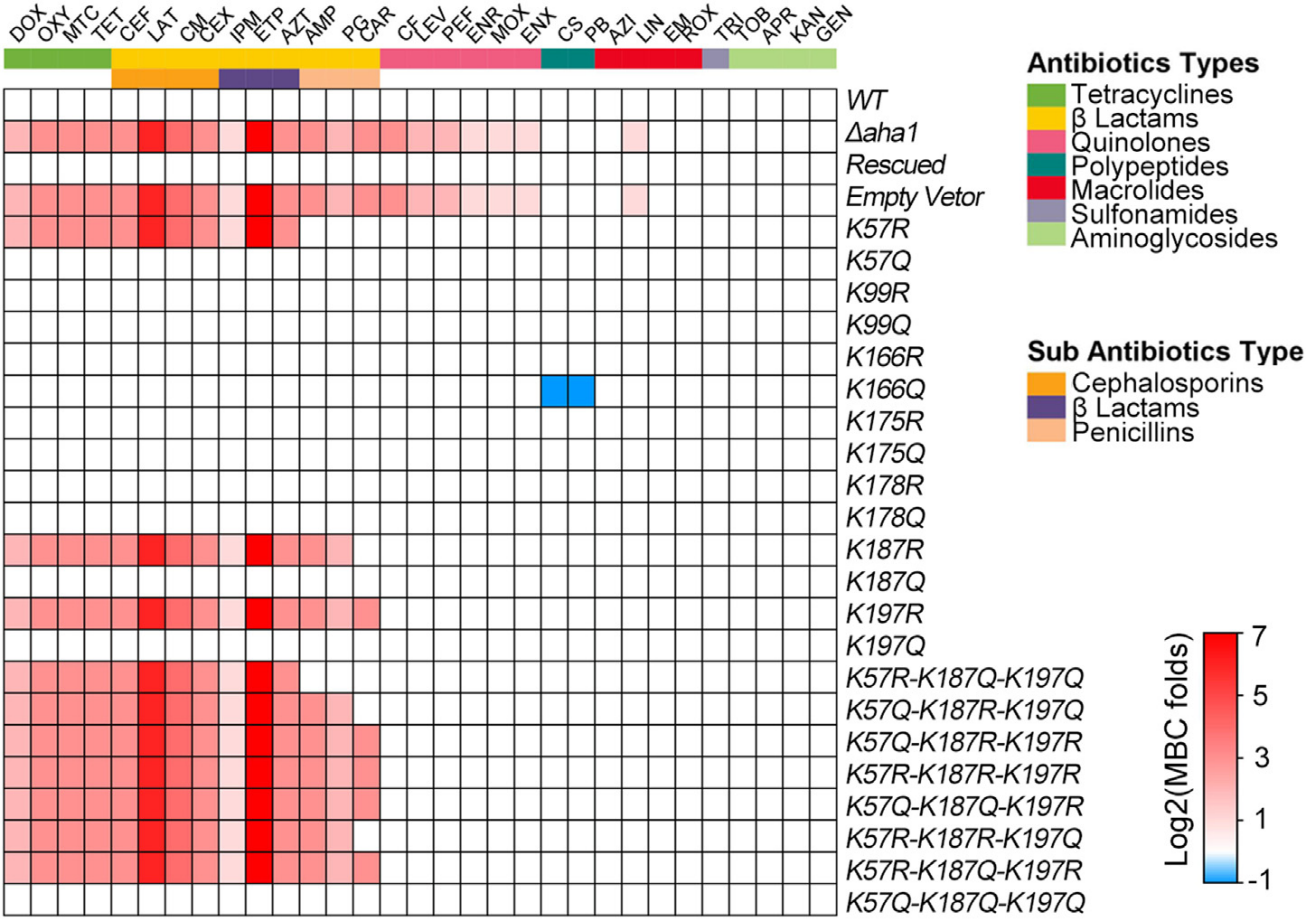
**Heatmap displays antibiotic susceptibility results of *Δaha1* and site-directed mutagenesis derivatives for 31 antibiotics using the MBC plate method.** The *horizontal* coordinate represents the abbreviations of different antibiotics. The *vertical* coordinates represent different *aha1* derivatives. The different color areas on the *right* side indicate the corresponding category or subcategory of antibiotics. The fold changes (mutant/WT) of MBCs are shown on the *right* side (log2 scale). Color grading represents a series of fold changes for different antibiotics.

Based on the fact that Kac, the state-specific sites of Aha1 strongly affect *A. hydrophila* against OXY stress, we then asked whether this pattern works only for OXY antibiotic or multidrugs resistance. Thus, the antibiotic susceptibility of the aforementioned seven Aha1 derivatives were further measured against a total of 31 antibiotics in this study (Figs. 9 and S6). These 31 antibiotics could be classified into seven groups: tetracyclines, β-Lactams, quinolones, polypeptides, macrolides, sulfonamides, aminoglycosides according to bactericidal mechanism, and β-lactams, which could also be classified into three sub-groups (cephalosporins, β-lactams, and penicillins). First, the deletion of *aha1* did not affect the MBCs to aminoglycosides, sulfonamides, polypeptides, and most macrolides except for lincomycin, for which the MBC increased two-fold. Besides these, *Δaha1* increased the MBCs to all tested tetracyclines, β-Lactams, quinolones antibiotics by 2 to 128-fold, and its rescued strains successfully restored the antibiotic-sensitive phenotypes, indicating the important role of Aha1 during multidrugs uptake.

Moreover, the K57R, K187R, and K197R mutants of *aha1* derivatives increased the MBCs against all tested tetracyclines and two kinds of antibiotics in the subantibiotics group (cephalosporins and β-lactams) to the same MBC folds as *Δaha1*. Although it belongs to β-lactams as well, it is a surprise that the K57R mutant did not affect the MBCs to penicillins, including AMP, PG, and CAR and that the K187R mutant did not affect the MBCs to CAR. Also surprising was the finding that K166Q decreased the MBCs to polypeptides (CS and PB) by two-fold, whereas other Aha1 mutants, including *Δaha1*, did not affect polypeptides susceptibility, indicating the complicated Kac regulation of multidrugs resistance. Moreover, the multiple site-directed mutagenesis generated K57, K187, and K197 mutants displayed the same resistance behaviors as their corresponding single site-directed mutagenesis mutants. Therefore, the multiple site-directed mutagenesis mutants did not reveal a superposition effect for antibiotic resistance.

## Discussion

The bacterial antibiotic resistance has seriously threatened human public health. With the emergence of super drug-resistant strains globally, it was anticipated that multidrug resistance will lead to the deaths of 10 million people each year worldwide by 2050 (33). Therefore, we must find an efficient way to deal with drug-resistant strains. Currently, there are many ARGs that have been reported to relate to bacterial TETs resistance and most of them are related to ribosomal protection, efflux pumps, modification of the drug target, and enzymatic alteration (34). For example, the acquisition of flavin-dependent monooxygenase-encoding genes termed *tet(X)* from the environmental microbiota can effectively inactivate TETs by enzymatic alteration mechanism, which confer high-level resistance to all TETs (34). For another example, the deletions and site-directed mutagenesis in *E. coli* OmpF and OmpC resulted in reducing permeability to various antibiotics including TETs (35, 36). However, although various new antibiotics have been discovered or invented, the current medical antimicrobial treatment, unfortunately, appears to make no promising progress, largely due to the poor understanding of the complex bacterial antibiotic resistance mechanisms. That pushed us to seek novel drug targets or clinical treatment tactics beyond the traditional well-known resistance mechanisms. Kac modification of proteins should be an ideal antibiotic resistance pattern based on at least two basic reasons: (1) Protein Kac modification is well distributed and plays important roles in diverse fundamental biological functions. Therefore, the AMR protein may change its structure and then switch the active site with antibiotics or affect its binding to DNA or RNA to regulate the transcription of a cluster of AMR genes; (2) The Kac modification is reversible and dynamical. These are attractive properties for Kac because the modified protein could adjust its structure or characters instantaneously according to the surrounding signals. If true, it is a smart strategy for bacteria to pay the lowest cost to survive in toxic environments. In addition, if the impact of PTMs on antibiotic resistance is common, the current clinical detection and research technologies that largely focus on ARG detection need to pay more attention to the PTM state of AMR proteins as well in the future. However, although several types of research pointed out the probable roles of the Kac on antibiotic resistance, direct evidence was still lacking until now. Meanwhile, *A. hydrophila* is a common bacterium that is well distributed in the world's freshwater environments. It is also a famous fish pathogen in commercial fisheries and an opportunistic pathogen in animal infection. Although more and more cases of multidrug resistance strains of this pathogen are being reported in clinical hospitals and aquaculture, the antibiotic resistance mechanism and prevention strategies are still poorly understood (37, 38). Therefore, we decided to explore the antibiotic-resistant role of Kac protein in *A. hydrophila* in this study. Using MS combined with an affinity enrichment method, we compared the differential expression in whole protein and Kac peptide levels between OXY-sensitive and OXY-resistant strains of the fish pathogen *A. hydrophila*.

A total of 4122 Kac peptides were identified and 98 upregulated and 628 downregulated in OXY^R^ strain in this study. The further motif analysis showed that at least five motifs were enriched in the altered Kac peptides that was different with the motifs enriched from total identified Kac peptides. Thus, it looks like that the antibiotic resistance condition may trigger a series of unknown acetyltransferases or deacetylases to regulate the drug resistance related to Kac sties in specific patterns. However, based on the facts that there are not confident evidences linking acetyltransferases and deacetylases to specific motifs and the chemical acetylation by acetyl-phosphate determinates global Kac in bacterial metabolism, the Kac motifs identified in this study may simply reflect the altered metabolism in OXY^R^ strain (39).

Many studies have revealed the behaviors of TET-resistant strains or sensitive strains under TET stress using proteomics methods in different bacterial species. These findings can be summarized as follows. First, the expression of several ribosomal subunits was significantly increased, which may be helpful to relieve the stress from TET antibiotics attacking a ribosomal site (40). Second, several iron homeostatic–related proteins, such as nonribosomal peptide synthetase A0KL39 and A0KL37, ferrichrome receptor A0KQZ1, colicin I receptor A0KQ46, and TonB-dependent siderophore receptor A0KJP9, were significantly increased as well. Although the detailed role of these proteins in antibiotic resistance is still unclear, the iron homeostasis affects antibiotic-mediated cell death in *Pseudomonas* spp., and the perturbation of the iron homeostasis process could promote the evolution of antibiotic resistance in *E. coli* and *A. hydrophila* (41, 42). Third, the proteins related to energy generation metabolic pathways were largely downregulated in the OXY^R^ strain. Previous studies have illustrated the important role of reducing bacterial metabolism to achieve antibiotic resistance, and our current proteomics data further confirmed it (43). Therefore, reprogramming intracellular metabolic homeostasis by boosting bacterial metabolism is a promising bacteriostatic strategy (44, 45). Finally, outer membrane proteins that may influx or pump the antibiotics out of the cell as a porin or channel play an important role in the antibiotic resistance (29). At least seven outer membrane proteins (A0KQ46, A0KJP9, A0KQZ1, A0KFM6, A0KFM8, SecB, and Aha1) were significantly altered in OXY^R^ in this study. In our previous research, the loss of A0KQ46 (*AHA_3963*), A0KJP9 (*AHA_1969*), and A0KQZ1 (*AHA_4275*) did not affect the antibiotic susceptibilities of *A. hydrophila* against TETs, including the OXY antibiotic. However, the deletion of *aha1* (*AHA_2785*) did significantly increased the MBC of *A. hydrophila* four to eight fold to OXY and TET (15). These results indicate that Aha1 may play an important role in OXY antibiotic resistance as an outer membrane porin channel.

We further confirmed the OXY resistance role of Aha1 by plate MBC assay. Aha1 is an outer membrane protein in *A. hydrophila* ATCC 7966, sharing 27% identities with OmpF, 29% with OmpC, and 28% with PhoE in *E. coli*, and belongs to the Gram-negative porin family. The deleted or mutant *E. coli* OmpF and PhoE exhibit a characteristic decrease in multidrug susceptibilities, such as β-lactams, ceftazidime, and ampicillin (46, 47). Therefore, Aha1 may act as a porin to control the entry of certain antibiotics into a cell.

Based on the diverse biological functions of proteins PTMs, we then focused on seeking the connection between lysine Kac modification and bacterial antibiotic resistance. In the Kac peptide level of this study, a large number of Kac modification peptides (accounting for 40.1% in all altered Kac peptides) were downregulated in the OXY^R^ strain. Previous studies have documented that the Kac level strongly intermediates intracellular energy and central carbon metabolic flux (7, 48). The lower acetylation level of various enzymes in *Salmonella* favors the glyoxylate pathway and gluconeogenesis (49). Meanwhile, the inactivation of the TCA cycle or energy generation–related metabolism was a common strategy for bacterial antibiotic resistance or tolerance against various antibiotics (50, 51). Therefore, this study's lower Kac level in the OXY^R^ strain indicates that bacteria may depress the central carbon metabolism against antibiotics *via* downregulating the intracellular Kac level.

Further analysis showed that at least 71 Kac peptides from 39 proteins belonging to different resistance mechanism classifications in the CARD database were altered in the OXY^R^ strain, suggesting that the Kac modification on AMR proteins may affect their roles in antibiotic resistance. We were then interested in the impact of Aha1 Kac modification on OXY resistance because Aha1 has seven downregulated Kac sites in the OXY^R^ strain, and the deletion of the *aha1* gene significantly increased the antibiotic resistance against OXY. Interestingly, the K57R, K187R, and K197R mutants significantly increased their MBCs to OXY eight fold, similar to the MBC of *Δaha1* to OXY. These results indicate that Aha1 controlled the porin permeability for OXY uptake mediated by Kac or deacetylation modification, and the OXY accumulation assay confirmed this hypothesis.

Based on the fact that the Aha1 homolog in the CARD database is classified as a general bacterial porin with reduced permeability to β-lactams, we hypothesized that the Aha1 and its Kac states might affect bacterial multidrug resistance in *A. hydrophila*. Therefore, we further measured the MBCs of these derivatives to a total of seven types of antibiotics on a large scale. To our surprise, the *Δaha1* and its K57R, K187R, and K197R mutants increased their MBCs up to 128-fold to many kinds of antibiotics, including tetracyclines and β-Lactam antibiotics; notably, for the resistance to oxacephem (RAO) and ETP, the MBCs were 64- and 128-fold, respectively. These results further confirmed that Aha1 and its Kac states were involved in bacterial multidrug resistance. Interestingly, the effects of the corresponding Aha1 derivatives (K57R, K187R, or K197R) were the same as *Δaha1*, whether as single point or multipoint mutants, indicating that there are no synergistic or superposition effects among them. Moreover, we also observed that *Δaha1* increased resistance to quinolone antibiotics but showed no differences among the series of Aha1 site-directed mutagenesis–generated mutants, suggesting the existence of other active sites in Aha1 besides the K57, K187, and K197 sites that are involved in the uptake of some specific antibiotics.

According to the Aha1 3D predicted structure, K57R is located on loop1, K187R and K197R on loop4. All of them are in the extracellular pore vestibule. Although K175 and K178 are near or located in loop4, both were more preferred to the pore's edge. Thus, the external K57, K187, and K197 residues may be involved in regulating the Aha1 pore size against antibiotics, and the Kac status of these three sites may play an important regulative role in it. Some research data supported this hypothesis. In the MSA analysis among *E. coli* OmpF, OmpC, PhoE, and *A. hydrophila* Aha1, the K57, K187, and K197 residues of Aha1 were not evolutionarily conservative residues, probably because the extracellular part of the protein undergoes more rapid mutational alterations to adapt to environments (32). However, using X-ray crystallography, Brigitte *et al*. predicted there are several hydrogen bonds between ampicillin COO^-^ and OmpF R167 and R168 residues (corresponding to the K197 range in Aha1), ampicillin NH3^+^ and OmpF Y32 residue (corresponding to the nearby of K57 in Aha1), that is helpful for ampicillin to bind to the extracellular pore vestibule of OmpF, oriented perpendicular to the pore axis. Moreover, the different charge features of β-Lactam antibiotics (zwitterionic AMP, anionic ETP, and di-anionic CAR) display different binding patterns with OmpF for antibiotics uptake (52). In our present study, the antibiotic resistance properties in Aha1 mutant porins suggest the Kac states of K57, K187, and K197 sites located at the extracellular pore vestibule of Aha1 are involved in the uptake of specific types of antibiotics, especially for tetracyclines and β-Lactam antibiotics.

Aha1 derivatives also showed different antibiotic resistance patterns to three penicillin antibiotics. That is, K57R had no effect on AMP, PG, and CAR, and K187R did not affect CAR. Although there are still no X-ray crystallography data to provide more robust evidence, the binding interactions among these lysine residues and the charges of these β-Lactam antibiotics may decide the target antibiotic uptake or not. Besides these mutants, the antibiotic sensibility of the K166Q Aha1 mutant was increased, while the WT and its deleted strains displayed no difference to polypeptides (CS and PB), which is a reminder that some AMR proteins could regulate bacterial antibiotic resistance by PTMs alone that escape the popular gene sequencing technologies used in the clinic.

Overall, our current data provided evidence to better understand how the Kac modification states on the extracellular loops of Aha1 porin affect outer membrane permeability and then trigger bacterial antibiotic resistance and may be helpful in the development of new antibiotic therapy strategies. In addition, although K to R (mimicking lack of Kac) and K to Q (mimicking presence of Kac) are useful models that have been well-used in many recent researches, the mutants remain imperfect since they are not real corresponding lysine modifications. That may partial explain some negative results obtained with these mutants.

## Data availability

Raw mass spectrometry proteomics data for whole cell proteins and Kac peptides have been deposited to the ProteomeXchange Consortium *via* the PRIDE partner repository with the dataset identifier PXD028447.

## Supplemental data

This article contains supplemental data.

## Conflict of interest

The authors declare no competing interests.
