# Supplementary material for: The Lysine Acetylation Modification in the Porin Aha1 of Aeromonas hydrophila Regulates the Uptake of Multidrug Antibiotics
Source: Mol Cell Proteomics. 2022 May 21;21(9):100248. doi: 10.1016/j.mcpro.2022.100248 (PMC9386498; doi:10.1016/j.mcpro.2022.100248)
Supplement: Supplementary Table S1 [file mmc1.docx]

**Supplementary Table S1: The primer pairs used in this study**

| REAGENT or RESOURCE | SOURCE | IDENTIFIER |
| --- | --- | --- |
| Oligonucleotides |  |  |
| Δ*aha1*+paha1-57R-F: 5’-CGCTGCATCTGGCTACAGGGATGTTGACG-3’ | This study | N/A |
| Δ*aha1*+paha1-57R-R: 5’-CTGTAGCCAGATGCAGCGGTAGAGTCAGCG-3’ | This study | N/A |
| Δ*aha1*+paha1-57Q-F: 5’-CGCTGCATCTGGCTACCAGGATGTTGACG-3’ | This study | N/A |
| Δ*aha1*+paha1-57Q-R: 5’-GGTAGCCAGATGCAGCGGTAGAGTCAGCG-3’ | This study | N/A |
| Δ*aha1*+paha1-99R-F: 5’-GCTGAAAACTCCGCCAACAGGTTTGACTCCC-3’ | This study | N/A |
| Δ*aha1*+paha1-99R-R: 5’-CTGTTGGCGGAGTTTTCAGCAGAAACTTG-3’ | This study | N/A |
| Δ*aha1*+paha1-99Q-F: 5’-TTCTGCTGAAAACTCCGCCAACCAGTTTGACTCCC-3’ | This study | N/A |
| Δ*aha1*+paha1-99Q-R: 5’-GGTTGGCGGAGTTTTCAGCAGAAACTTGCC-3’ | This study | N/A |
| Δ*aha1*+paha1-166R-F: 5’-CGGCGGCTTCAAAGGCAGACTGTCCTATCAG-3’ | This study | N/A |
| Δ*aha1*+paha1-166R-R: 5’-CTGCCTTTGAAGCCGCCGTAGGTGTTGGAG-3’ | This study | N/A |
| Δ*aha1*+paha1-166Q-F: 5’-CGGCGGCTTCAAAGGCCAACTGTCCTATCAG-3’ | This study | N/A |
| Δ*aha1*+paha1-166Q-R: 5’-GGCCTTTGAAGCCGCCGTAGGTGTTGGAG-3’ | This study | N/A |
| Δ*aha1*+paha1-175R-F: 5’-TATCAGACCAACGACGACAGGGCCGTCAAG-3’ | This study | N/A |
| Δ*aha1*+paha1-175R-R: 5’-CTGTCGTCGTTGGTCTGATAGGACAGTTTG-3’ | This study | N/A |
| Δ*aha1*+paha1-175Q-F: 5’-TATCAGACCAACGACGACCAGGCCGTCAAG-3’ | This study | N/A |
| Δ*aha1*+paha1-175Q-R: 5’-GGTCGTCGTTGGTCTGATAGGACAGTTTG-3’ | This study | N/A |
| Δ*aha1*+paha1-187R-F: 5’-CTGACGTAGGTCAGGGTATCAGAGAAACTGCC-3’ | This study | N/A |
| Δ*aha1*+paha1-187R-R: 5’-CTGATACCCTGACCTACGTCAGTAACCTT-3’ | This study | N/A |
| Δ*aha1*+paha1-187Q-F: 5’-CTGACGTAGGTCAGGGTATCCAAGAAACTGCC-3’ | This study | N/A |
| Δ*aha1*+paha1-187Q-R: 5’-GGATACCCTGACCTACGTCAGTAACCTTG-3’ | This study | N/A |
| Δ*aha1*+paha1-197R-F: 5’-GTTTACGGTGCCGATGTGAGGCGTAACTAC-3’ | This study | N/A |
| Δ*aha1*+paha1-197R-R: 5’-CTCACATCGGCACCGTAAACGGCAGTTTC-3’ | This study | N/A |
| Δ*aha1*+paha1-197Q-F: 5’-GTTTACGGTGCCGATGTGCAGCGTAACTAC-3’ | This study | N/A |
| Δ*aha1*+paha1-197Q-R: 5’-GCACATCGGCACCGTAAACGGCAGTTTC-3’ | This study | N/A |
| Δ*aha1*+paha1-F: 5’-GTCGACGGTATCGATAAGCTTGATACCTGCAACTGGCAACCG-3’ | This study | N/A |
| Δ*aha1*+paha1-R: 5’-CGCTCTAGAACTAGTGGATCCCTAATGGTGATGGTGATGATGGAAG  TTGTATTGCAGGGCAACAGT-3’ | This study | N/A |
| *aha1*-F: 5’-CCTCGAGGTCGACGGTATCG-3’ | This study | N/A |
| *aha1*-R: 5’-GGCGGCCGCTCTAGAACTAG-3’ | This study | N/A |
| *aha1*-P1: 5’-CATGAATTCCCGGGAGAGCTCTCAGTGTCTTGGCTATCTCGGC-3’ | This study | N/A |
| *aha1*-P2: 5’-AGGCTGGACACTAAGTCGTTGTTATTCCCTGTG-3’ | This study | N/A |
| *aha1*-P3: 5’-AACGACTTAGTGTCCAGCCTCTGCGCTGATT-3’ | This study | N/A |
| *aha1*-P4: 5’-CGATCCCAAGCTTCTTCTAGATCTTGCGATAGAGCGCGG-3’ | This study | N/A |
| *aha1*-P5: 5’-TATCCCGGCTCTGTTTGCATC-3’ | This study | N/A |
| *aha1*-P6: 5’-GAAGTTGTATTGCAGGGCAACAG-3’ | This study | N/A |
| *aha1*-P7: 5’-TTCTTCTGGGTGAGCCGTG-3’ | This study | N/A |
| *aha1*-P8: 5’-ATCACTTGCTGCAGCGCCT-3’ | This study | N/A |
| *AHA_1969*-P1: 5’-CGATCCCAAGCTTCTTCTAGACATCCTGATCACCAGCCTGC-3’ | This study | N/A |
| *AHA_1969*-P2: 5’-CAAACAGAGGTTATGGGGTCCG-3’ | This study | N/A |
| *AHA_1969*-P3: 5’-GACCCCATAACCTCTGTTTGATTACAAGCTGTAAACCCCGC-3’ | This study | N/A |
| *AHA_1969*-P4: 5’-CATGAATTCCCGGGAGAGCTCAGAGCAGCAGGAAGTTGGAGAG-3’ | This study | N/A |
| *AHA_1969*-P5: 5’-AGTTGCAGCCTGGCGTATTTC-3’ | This study | N/A |
| *AHA_1969*-P6: 5’-CAGCTTGTAATCCACGCCAGC-3’ | This study | N/A |
| *AHA_1969*-P7: 5’-CTGCAGATGTTCGGTCACTTC-3’ | This study | N/A |
| *AHA_1969*-P8: 5’-GCGACCCCAGATTCCAGAAAC-3’ | This study | N/A |
| *AHA_3793*-P1: 5’-CGATCCCAAGCTTCTTCTAGAAAGCGGCTGATGCTGTAGATG-3’ | This study | N/A |
| *AHA_3793*-P2: 5’-AGGATCACTGCATGTTGAGCCTCTGCAATTTC-3’ | This study | N/A |
| *AHA_3793*-P3: 5’-GCTCAACCAGTGATCCTCAAGCGTGGC-3’ | This study | N/A |
| *AHA_3793*-P4: 5’-CATGAATTCCCGGGAGAGCTCTCAAGCCGGTGTCCCTCTC-3’ | This study | N/A |
| *AHA_3793*-P5: 5’-GCTGGTTTCAGGCTTGCTTG-3’ | This study | N/A |
| *AHA_3793*-P6: 5’-CACTGCTGAACTTCCGAGATC-3’ | This study | N/A |
| *AHA_3793*-P7: 5’-CTGAACGGCTTGATGTCGTC-3’ | This study | N/A |
| *AHA_3793*-P8: 5’-GACGCAGCTGCACTACTACTC-3’ | This study | N/A |
| *saPD*-P1: 5’-CATGAATTCCCGGGAGAGCTCAGGTTTACCTTTGGCAACGCC-3’ | This study | N/A |
| *saPD*-P2: 5’-TTTGAGTGCTTCGCGAATGC-3’ | This study | N/A |
| *saPD*-P3: 5’-GCATTCGCGAAGCACTCAAAATTCAATCCCGTATCAGAGCGT-3’ | This study | N/A |
| *saPD*-P4: 5’-CGATCCCAAGCTTCTTCTAGAATCTGCGGATAGAAGAGGGCG-3’ | This study | N/A |
| *saPD*-P5: 5’-ACTGATGCCTCTGCTCGAC-3’ | This study | N/A |
| *saPD*-P6: 5’-TGGGCTCTTCCATGTTCAACG-3’ | This study | N/A |
| *saPD*-P7: 5’-CAACAGCGGCAACATCGAT-3’ | This study | N/A |
| *saPD*-P8: 5’-AGATATCCAGGGTGGAGAGC-3’ | This study | N/A |
| *oppD*-P1: 5’-CATGAATTCCCGGGAGAGCTCTTCTTCGGCCGCAATCTG-3’ | This study | N/A |
| *oppD*-P2: 5’-TTGTCAGTCCTTCCTGTCGATCCGATTCGC-3’ | This study | N/A |
| *oppD*-P3: 5’-TCGACAGGAAGGACTGACAAGAAACTGCTGCTG-3’ | This study | N/A |
| *oppD*-P4: 5’-CGATCCCAAGCTTCTTCTAGAAGAACTCATGGGGATAGCGGT-3’ | This study | N/A |
| *oppD*-P5: 5’-CGTCACGAAAGATCGATAAGGAG-3’ | This study | N/A |
| *oppD*-P6: 5’-CCCATTTAGTTCACTCCCTTG-3’ | This study | N/A |
| *oppD*-P7: 5’-TCATCATCGGCACTCTCTAC-3’ | This study | N/A |
| *oppD*-P8: 5’-CATCTCCTTCTGGATAGCTT-3’ | This study | N/A |
| *AHA_4275*-P1: 5’-CGATCCCAAGCTTCTTCTAGACGGATTATGGATCTCGGCC-3’ | This study | N/A |
| *AHA_4275*-P2: 5’-GCAGCTGCAGGTGATTCTTATTATTGGTGCCCGGTC-3’ | This study | N/A |
| *AHA_4275*-P3: 5’-TAAGAATCACCTGCAGCTGCTCTGATC-3’ | This study | N/A |
| *AHA_4275*-P4: 5’-CATGAATTCCCGGGAGAGCTCCACCACCATGGTGAAGGTGAC-3’ | This study | N/A |
| *AHA_4275*-P5: 5’-CATGACCGGGCACCAATAAT-3’ | This study | N/A |
| *AHA_4275*-P6: 5’-ATTACCAGCGGTAGTTGATGC-3’ | This study | N/A |
| *AHA_4275*-P7: 5’-TGCTCGGCAAGATCCTTGGC-3’ | This study | N/A |
| *AHA_4275*-P8: 5’-CTGATAGAGCCGATCCTCGG-3’ | This study | N/A |
| *fepA*-P1: 5’-CGATCCCAAGCTTCTTCTAGACGTTCACTTCTCCGCCATCC-3’ | This study | N/A |
| *fepA*-P2: 5’-CGGGTCATCAGAAGTTGTGTTATCATGTTGGCCTGAACTCT-3’ | This study | N/A |
| *fepA*-P3: 5’-ACACAACTTCTGATGACCCGCCG-3’ | This study | N/A |
| *fepA*-P4: 5’-CATGAATTCCCGGGAGAGCTCGCTGCTGTTCGATCTCGATCG-3’ | This study | N/A |
| *fepA*-P5: 5’-ATGCACCGCAACAAGCTGAC-3’ | This study | N/A |
| *fepA*-P6: 5’-TTGTAGGTCATGCCCAGGGTG-3’ | This study | N/A |
| *fepA*-P7: 5’-GACTGGTACCGTCAAGTTCT-3’ | This study | N/A |
| *fepA*-P8: 5’-TGGCCTACATCATCCAGTTG-3’ | This study | N/A |
| *AHA_0461*-P1: 5’-CGATCCCAAGCTTCTTCTAGACATCGTGTTTCTCGCCGC-3’ | This study | N/A |
| *AHA_0461*-P2: 5’-GGAGTTGCGTGTTGACTCTTGAAGAAG-3’ | This study | N/A |
| *AHA_0461*-P3: 5’-AAGAGTCAACACGCAACTCCCTCTGGCCAGCTTCAG-3’ | This study | N/A |
| *AHA_0461*-P4: 5’-CATGAATTCCCGGGAGAGCTCAGGCGAAGTTGGCCTGCT-3’ | This study | N/A |
| *AHA_0461*-P5: 5’-ATGCACACACCTCACCACCC-3’ | This study | N/A |
| *AHA_0461*-P6: 5’-CTGACGAAGTAGTCGTGATC-3’ | This study | N/A |
| *AHA_0461*-P7: 5’-GAAGCCCGTCGCTACCAGT-3’ | This study | N/A |
| *AHA_0461*-P8: 5’-CGTGCAGCTTGTTCCACATC-3’ | This study | N/A |
| *AHA_3063*-P1: 5’-CGATCCCAAGCTTCTTCTAGACTCTGTGAGGTGATGGCGGT-3’ | This study | N/A |
| *AHA_3063*-P2: 5’-GCCATGAGATTGAGTTCTCCTTCACTGAGTCGG-3’ | This study | N/A |
| *AHA_3063*-P3: 5’-GGAGAACTCAATCTCATGGCCAGAATGAAATTC-3’ | This study | N/A |
| *AHA_3063*-P4: 5’-CATGAATTCCCGGGAGAGCTCGAGATGACCTGCGCATCCC-3’ | This study | N/A |
| *AHA_3063*-P5: 5’-ATGAAACTGACGAAACGTGC-3’ | This study | N/A |
| *AHA_3063*-P6: 5’-CTACTTGGCGAAAACGGCAC-3’ | This study | N/A |
| *AHA_3063*-P7: 5’-TCACCGGCTCTTTGATGGAG-3’ | This study | N/A |
| *AHA_3063*-P8: 5’-CATTAGCCAGGTTTTCATGC-3’ | This study | N/A |
| *fklB*-P1: 5’-CGATCCCAAGCTTCTTCTAGAGTCAATGCCGACAACGTGC-3’ | This study | N/A |
| *fklB*-P2: 5’-ATCAATCAAGCCAGCCGGATGAGAAGTTGCTCCGGGTCC-3’ | This study | N/A |
| *fklB*-P3: 5’-ATCCGGCTGGCTTGATTGA-3’ | This study | N/A |
| *fklB*-P4: 5’-CATGAATTCCCGGGAGAGCTCAAAGCTTGCGGCTGTCCC-3’ | This study | N/A |
| *fklB*-P5: 5’-GTCCCAATACGACTCTATCG-3’ | This study | N/A |
| *fklB*-P6: 5’-TCACAGGATGTCCAGCAGC-3’ | This study | N/A |
| *fklB*-P7: 5’-CTGCGAGCGCATTCTGAGC-3’ | This study | N/A |
| *fklB*-P8: 5’-TCGATGCGCTGCCAGAAGTG-3’ | This study | N/A |
| *iscS*-P1: 5’-CGATCCCAAGCTTCTTCTAGATTGCATGCAGAGCAGGGG-3’ | This study | N/A |
| *iscS*-P2: 5’-GAACACCGATGTTTTTCTCCGTTACAGCCAGG-3’ | This study | N/A |
| *iscS*-P3: 5’-GGAGAAAAACATCGGTGTTCAACGGAATTAGC-3’ | This study | N/A |
| *iscS*-P4: 5’-CATGAATTCCCGGGAGAGCTCAGAAACGAGGAAACCCGTGC-3’ | This study | N/A |
| *iscS*-P5: 5’-ATGAAACTGCCTATTTACCTTG-3’ | This study | N/A |
| *iscS*-P6: 5’-ATGAAACTGCCTATTTACCTTG-3’ | This study | N/A |
| *iscS*-P7: 5’-ACTTGACTGATTTAGTCGGGTATG-3’ | This study | N/A |
| *iscS*-P8: 5’-AATGATCTTGACCCCGTCCTGCTCG-3’ | This study | N/A |
| CobB-32a-F: 5’-CTGATATCGGATCCGAATTCATGGTGCAGTCAGCGAAACAC-3’ | This study | N/A |
| CobB-32a-R: 5’-CTCGAGTGCGGCCGCAAGCTTTCAGGGACCGCGCATCTC-3’ | This study | N/A |
| AcuC-32a-F: 5’-GCTGATATCGGATCCGAATTCGTGCGACAAGGGGAGCCT-3’ | This study | N/A |
| AcuC-32a-R: 5’-CTCGAGTGCGGCCGCAAGCTTCTAGCCGTAGCGCTTTTTCG-3’ | This study | N/A |
| Aha1-28a-F: 5’-CAGCAAATGGGTCGCGGATCCATGAAAAAGACAATTCTGGCTATTGC-3’ | This study | N/A |
| Aha1-28a-R: 5’-CTCGAGTGCGGCCGCAAGCTTTTAGAAGTTGTATTGCAGGGCAAC-3’ | This study | N/A |
| *lpaha1*-P1: 5’-CGATCCCAAGCTTCTTCTAGACAGTGTCTTGGCTATCTCGGCT-3’ | This study | N/A |
| *lpaha1*-P2: 5’-GGACCAATGCCTACTGTAATTAAACACTAAG-3’ | This study | N/A |
| *lpaha1*-P3: 5’-TACAGTAGGCATTGGTCCAGCGTCTGCGCTGAT-3’ | This study | N/A |
| *lpaha1*-P4: 5’-CATGAATTCCCGGGAGAGCTCCTCTTGCGATAGAGCGCGG-3’ | This study | N/A |
| *lpaha1*-P5: 5’-TCTGGCTATTGCTATCCCGGC-3’ | This study | N/A |
| *lpaha1*-P6: 5’-GTTGTATTGCAGGGCAACAGTC-3’ | This study | N/A |
| *lpaha1*-P7: 5’-GTTGTATTGCAGGGCAACAGTC-3’ | This study | N/A |
| *lpaha1*-P8: 5’-CCACAGGATCTTGCAAAGG-3’ | This study | N/A |
| Δ*lpaha1*+lpaha1-F: 5’-GTCGACGGTATCGATAAGCTTGAAAAAAATGACCGTTGCACAG-3’ | This study | N/A |
| Δ*lpaha1*+lpaha1-R: 5’-CGCTCTAGAACTAGTGGATCCTTAATGGTGATGGTGATGATGGAAGTTGTATTGCAGGGCAACAGTCCACTC-3’ | This study | N/A |
