# Supplementary material for: The Lysine Acetylation Modification in the Porin Aha1 of Aeromonas hydrophila Regulates the Uptake of Multidrug Antibiotics
Source: Mol Cell Proteomics. 2022 May 21;21(9):100248. doi: 10.1016/j.mcpro.2022.100248 (PMC9386498; doi:10.1016/j.mcpro.2022.100248)

Figure S1


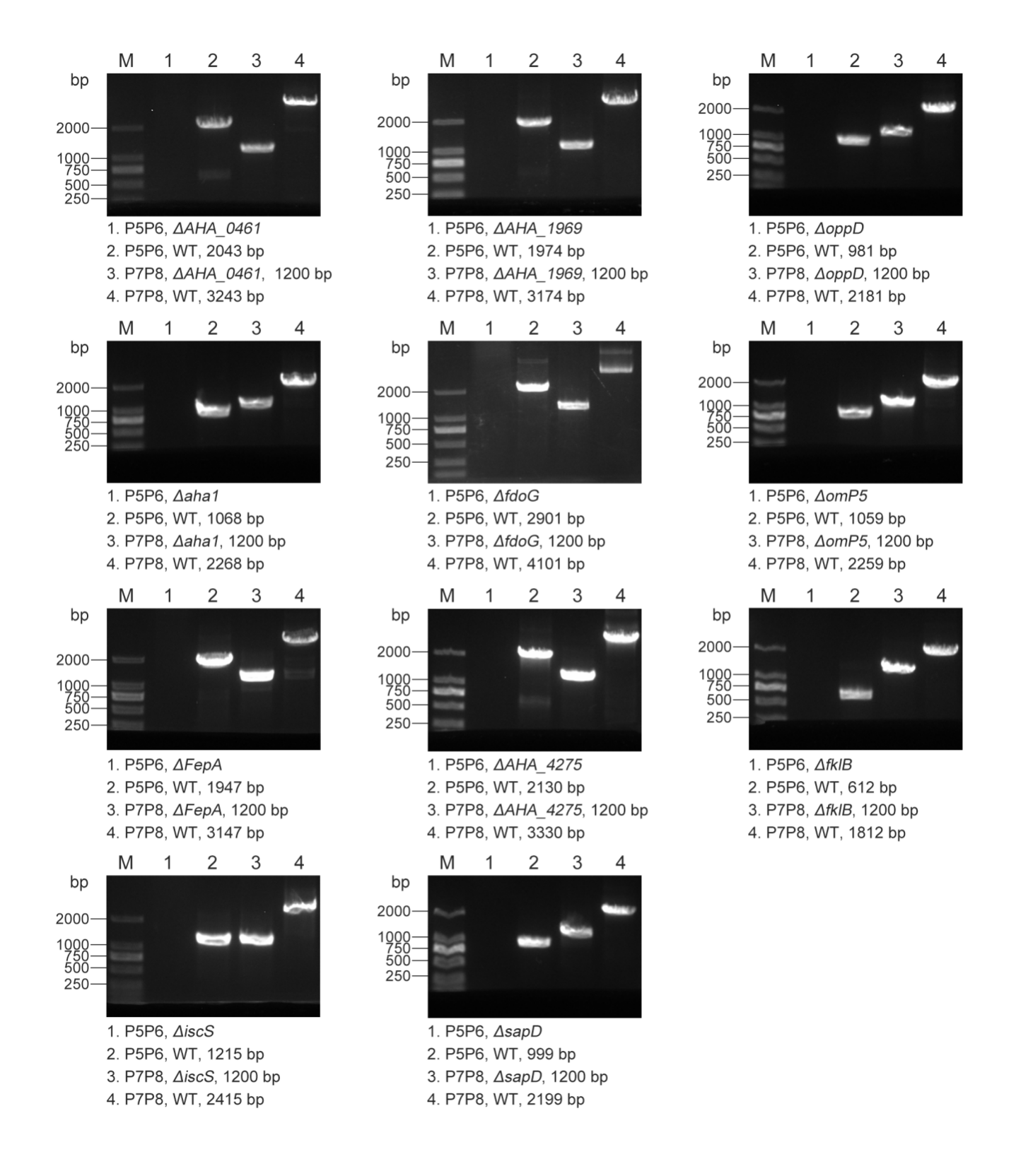


Figure S2


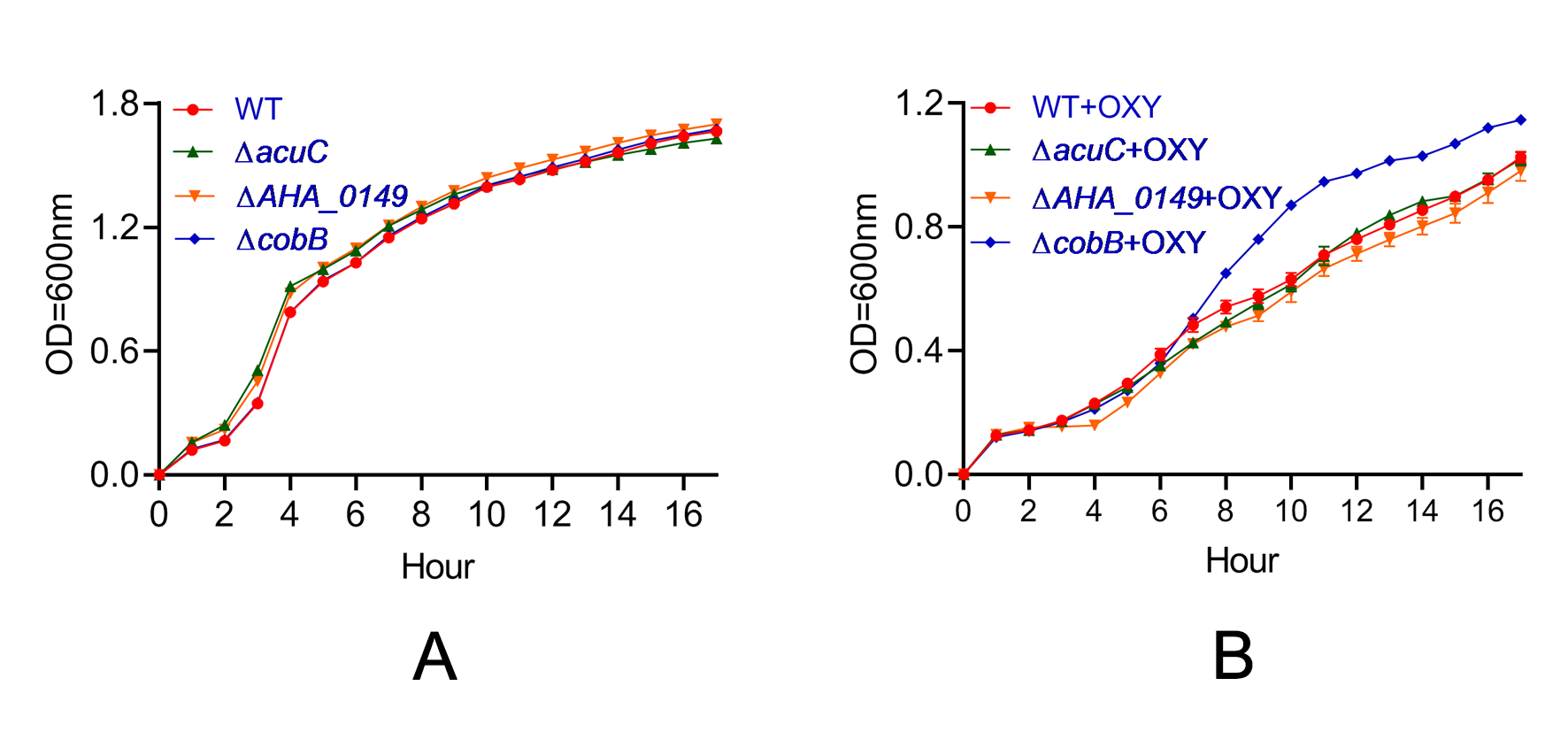


Figure S3


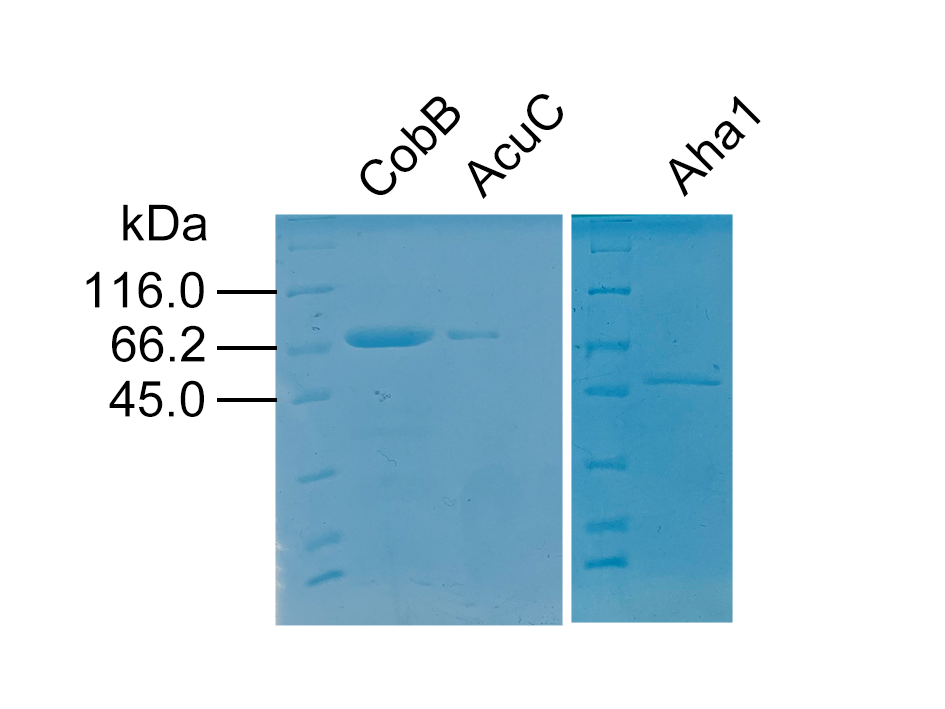


Figure S4


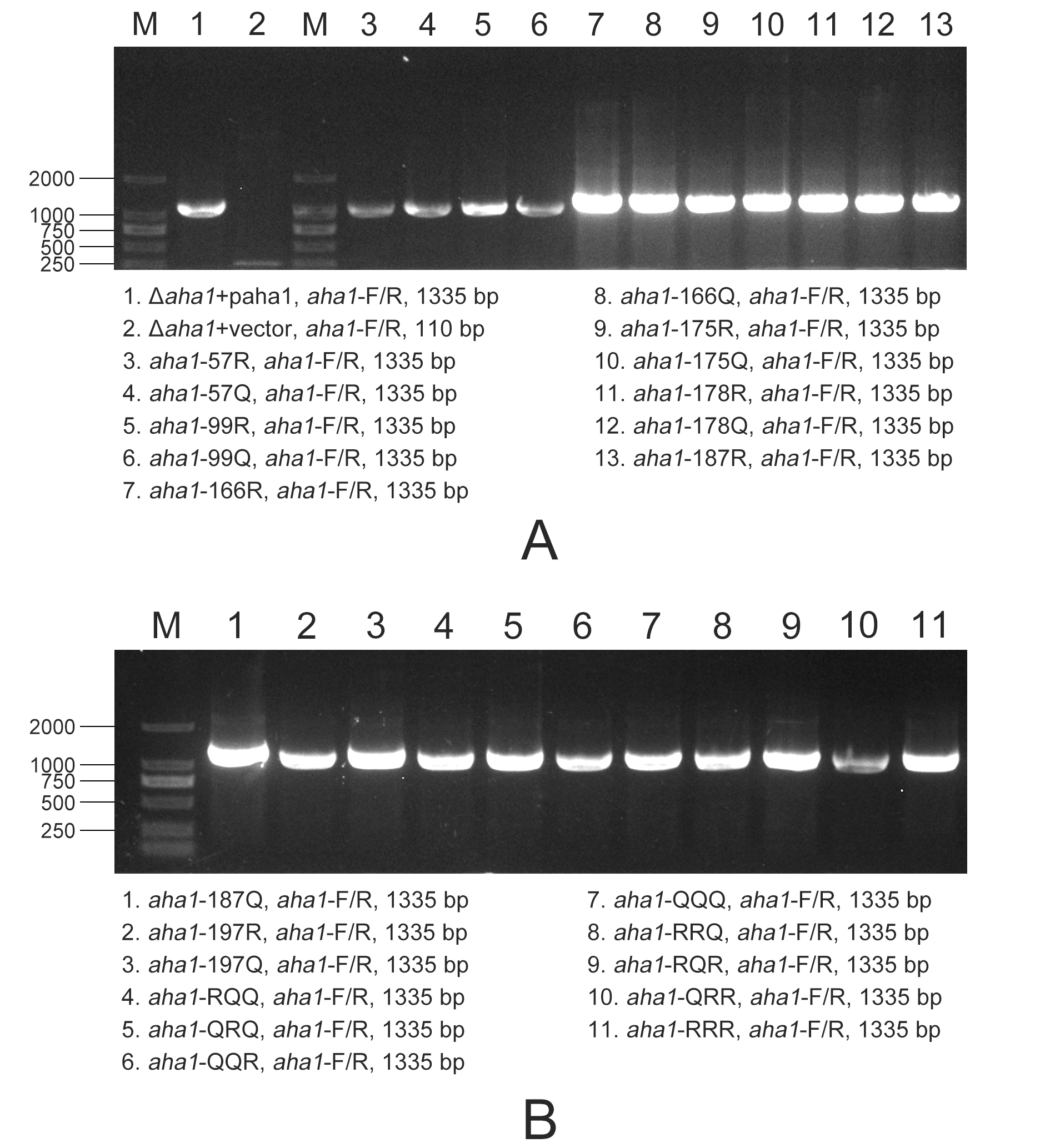


Figure S5


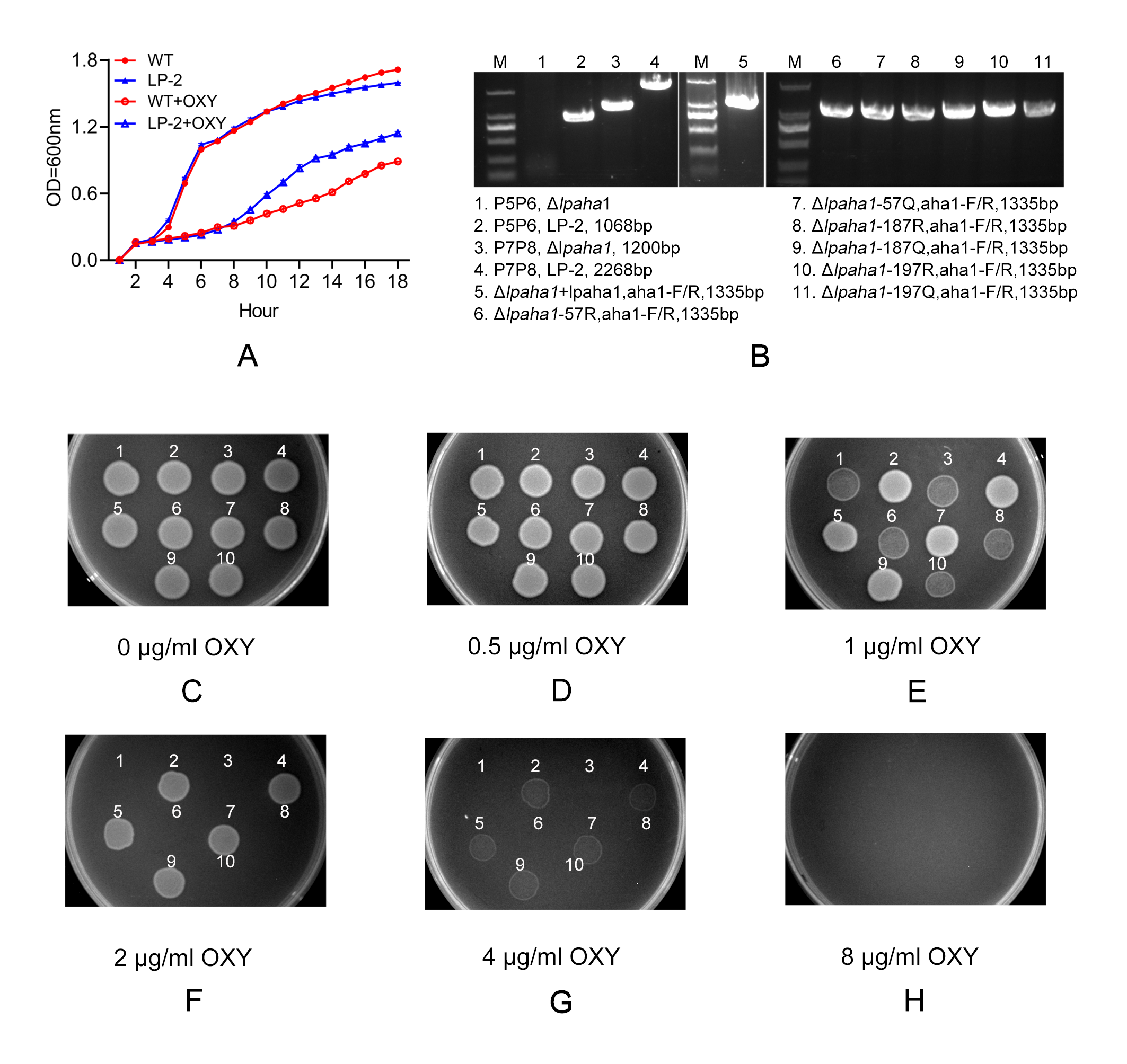


Figure S6

1. Tetracyclines

1.1 Doxycycline (DOX)


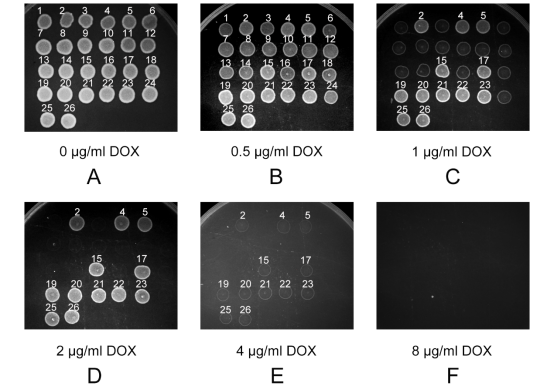


1.2 Oxytetracycline(OXY)


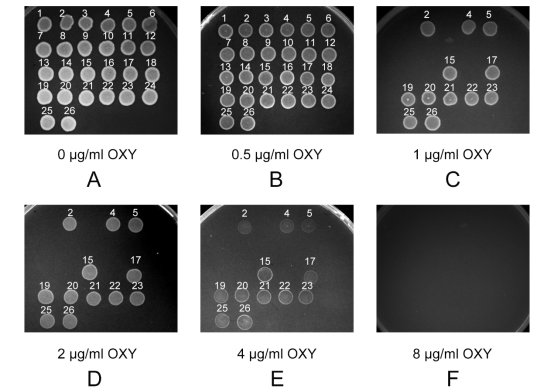


1.3 Metacycline (MTC)


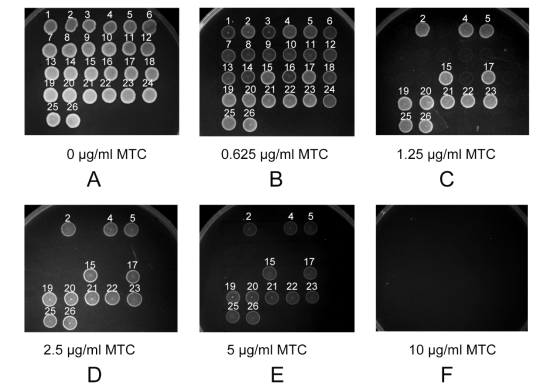


1.4 Tetracycline(TET)


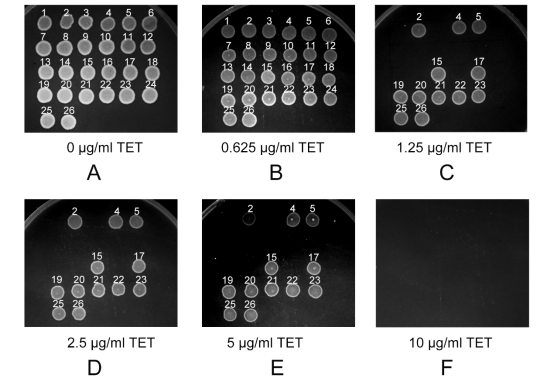


2. β-lactams

2.1 Ceftriaxone (CEF)


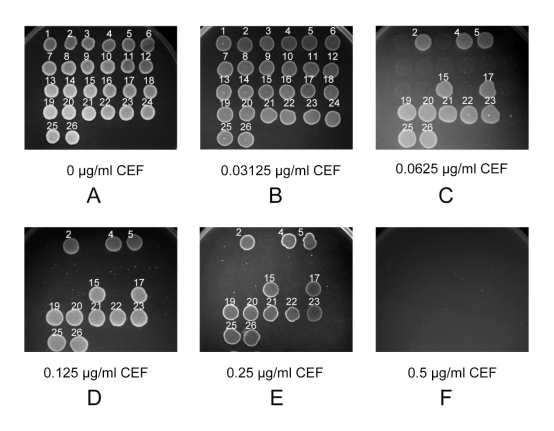


2.2 Latamoxef (LAT)


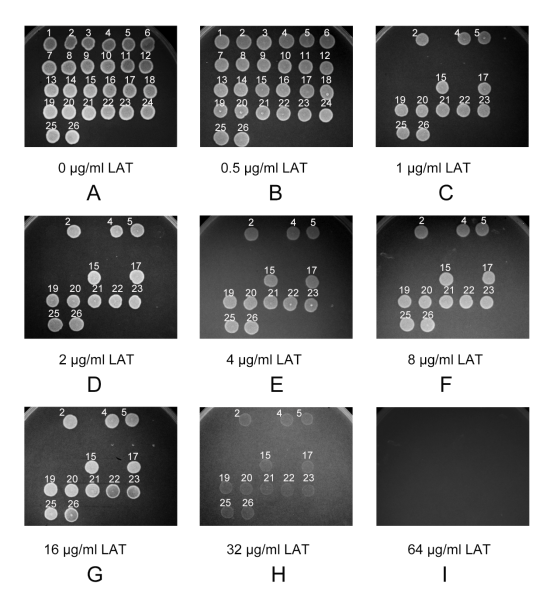


2.3 Cefamandole (CM)


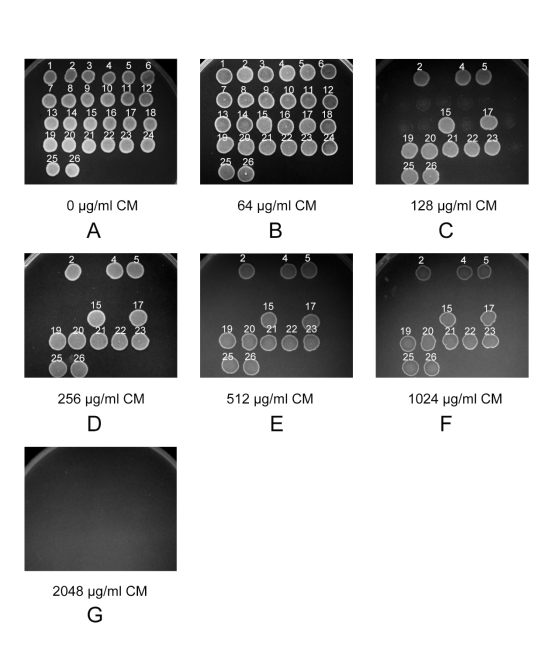


2.4 Cefazolin (CEX)


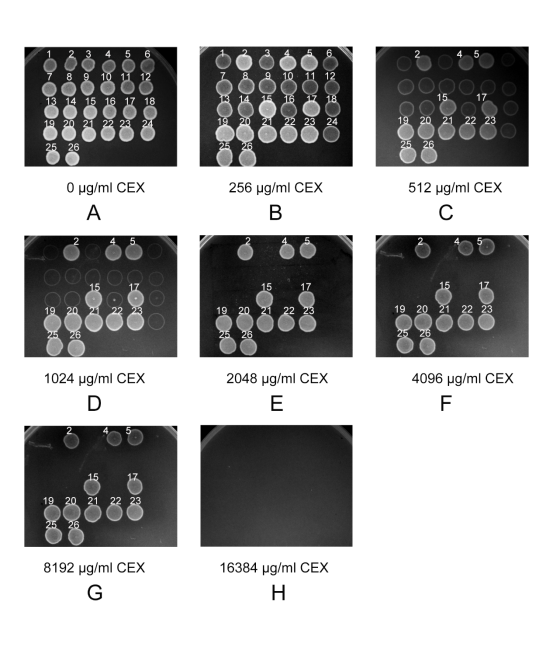


2.5 Imipenem (IPM)


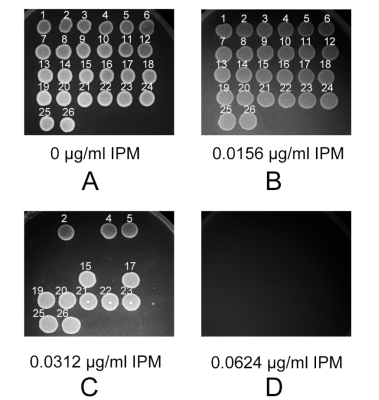


2.6 Ertapenem (ETP)


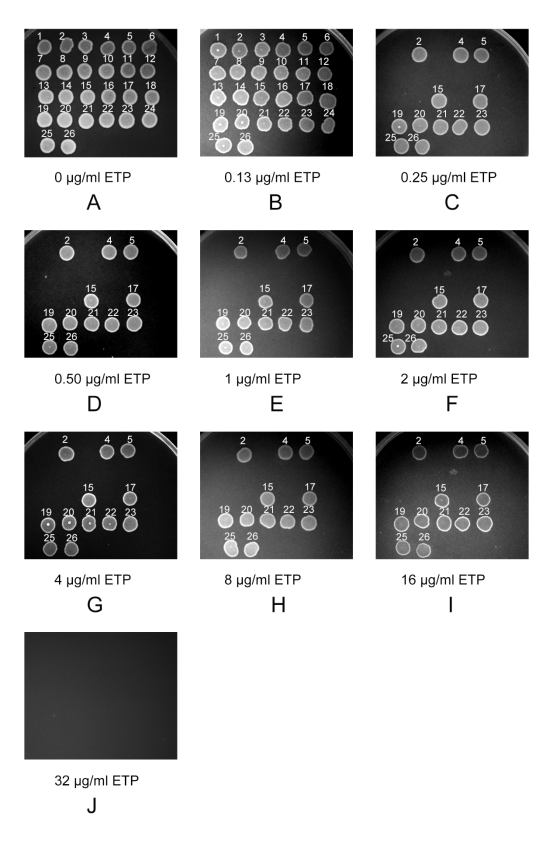


2.7 Aztreonam (AZT)


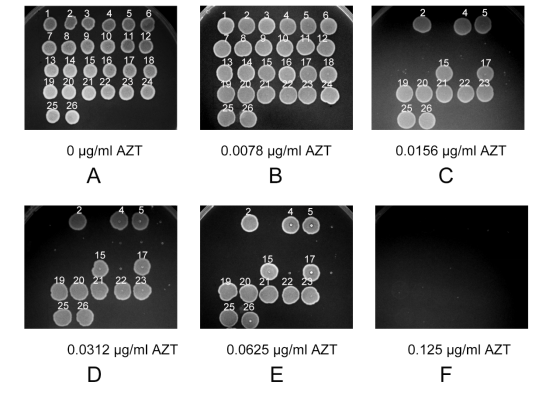


2.8 Ampicillin (AMP)


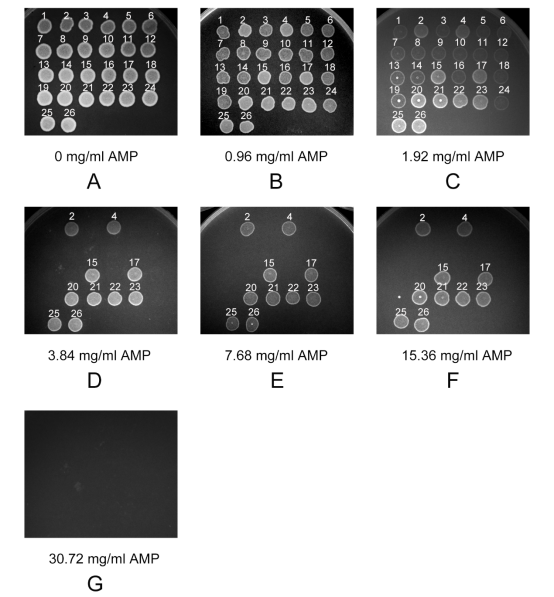


2.9 Penicillin G (PG)


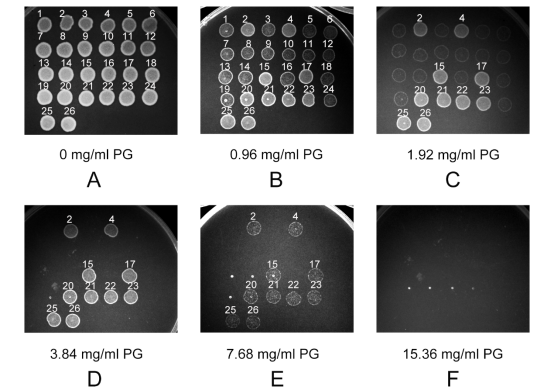
 2.10 Carboxybenzylpenicillin(CAR)


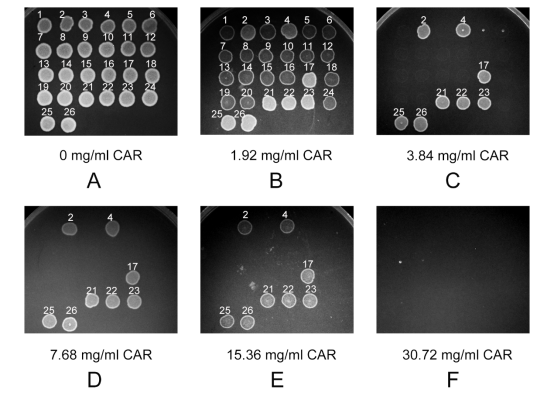


3. Quinolones

3.1 Ciprofloxacin (CF)


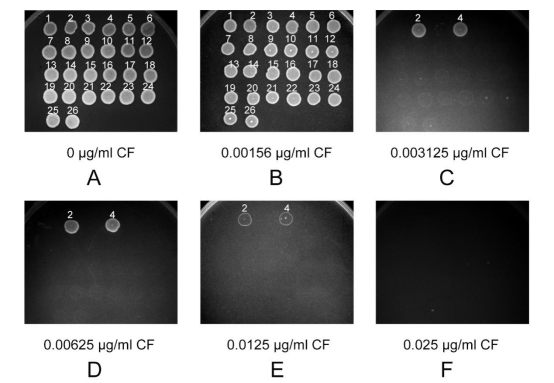


3.2 Levofloxacin(LEV)


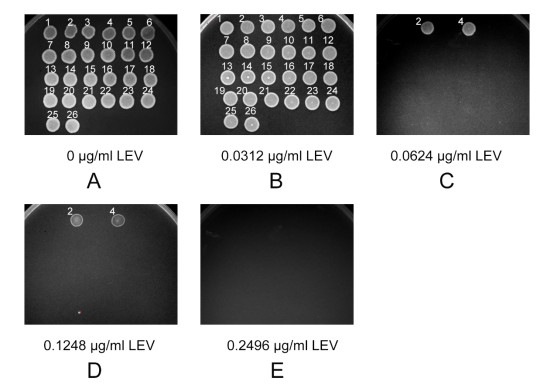


3.3 Pefloxacin (PEF)


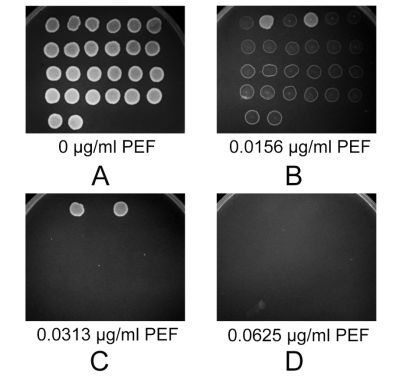


3.4 Enrofloxacin (ENR)


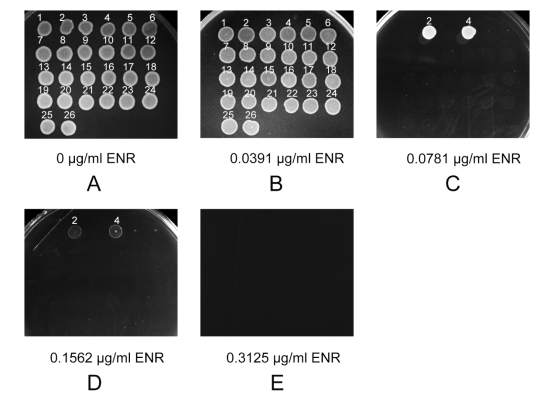


3.5 Moxifloxacin (MOX)


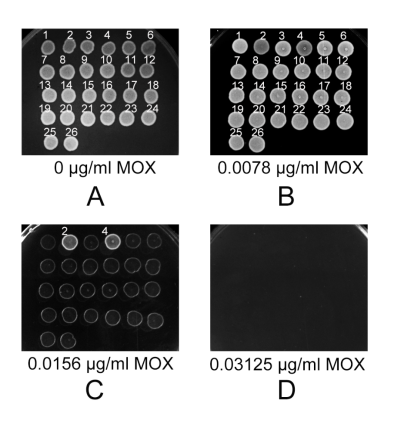


3.6 Enoxacin (ENX)


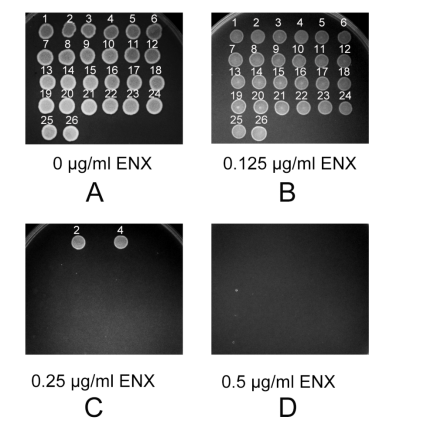


4. Polypeptides

4.1 Colistin sulfate (CS)


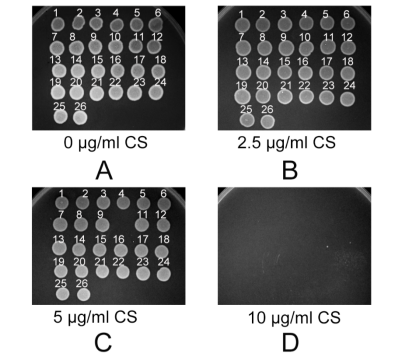


4.2 Polymyxin sulfate B (PB)


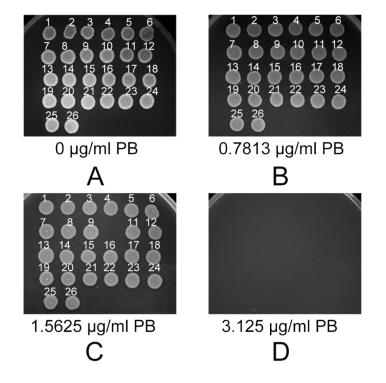


5. Macrolides

5.1 Azithromycin (AZI)


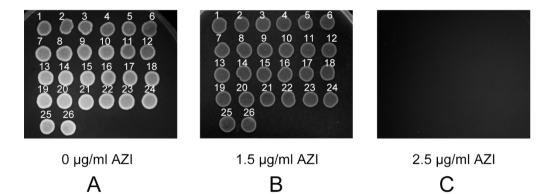


5.2 Lincomycin (LIN)


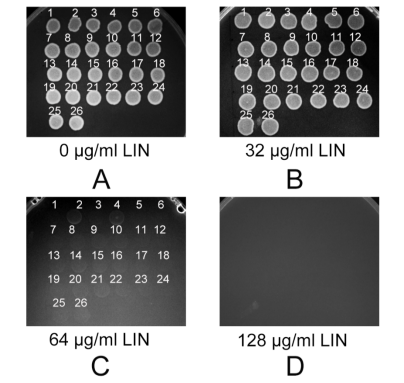


5.3 Erythromycin (EM)


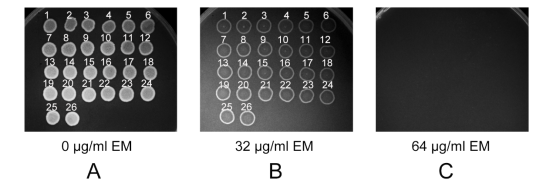


5.4 Roxithromycin (ROX)


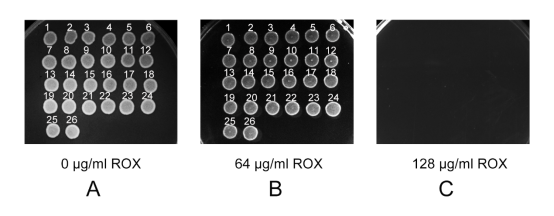


6. Sulfonamides

6.1 Trimethoprim (TRI)


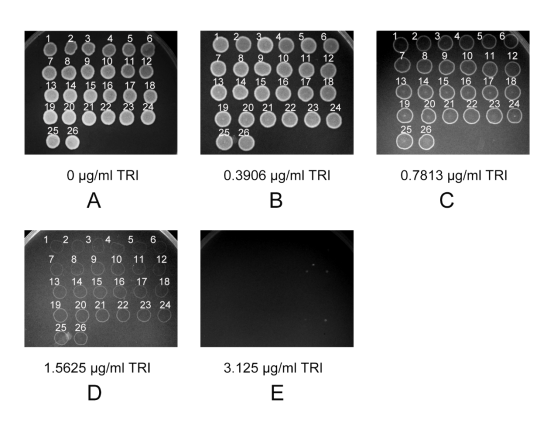


7. Aminoglycosides

7.1 Tobramycin (TOB)


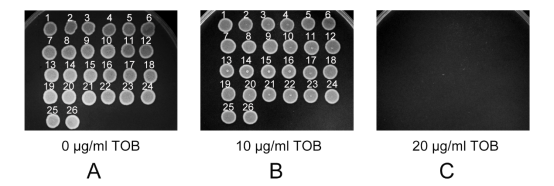


7.2 Apramycin (APR)


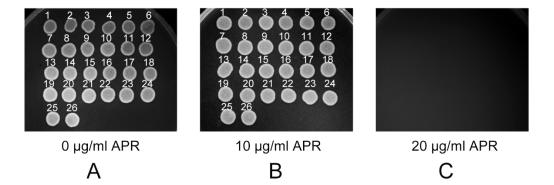


7.3 Kanamycin (KAN)


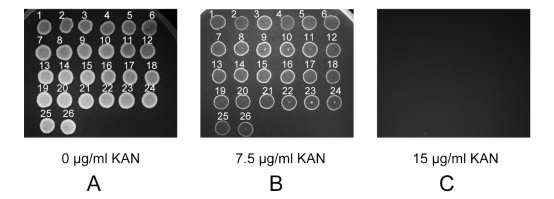


7.4 Gentamicin (GEN)


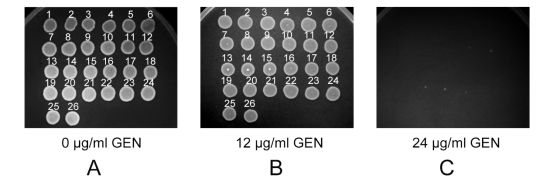

Supplement: Supplementary Figures [file mmc8.docx]
