# Supplementary material for: The Lysine Acetylation Modification in the Porin Aha1 of Aeromonas hydrophila Regulates the Uptake of Multidrug Antibiotics
Source: Mol Cell Proteomics. 2022 May 21;21(9):100248. doi: 10.1016/j.mcpro.2022.100248 (PMC9386498; doi:10.1016/j.mcpro.2022.100248)
Supplement: Supplementary Materials [file mmc9.docx]

**SUPPLEMENTARY MATERIALS**

**Figure S1. Construction and confirmation of the resistance gene mutant strains.** The fragments of DNA amplified using the primer pair P5/P6 (located upstream and downstream of the target gene) and P7/P8 (It is located 600bp upstream and downstream of the target gene) in the target mutants with WT strain as control, respectively, are displayed. M: DL2000 marker; 1: mutant strains, P5/P6 was used as primer; 2: WT, P5/P6 was used as primer; 3: mutant strains, P7/P8 was used as primer; 4: WT, P7/P8 was used as primer.

**Figure S2. *aha1* gene deleted and several site mutations affect OXY resistance in *A. hydrophila* LP-2 strain.** (A) Growth curves of LP-2 and WT with or without OXY stress; (B) Validation of *lpaha1* deletion strain and its site-directed mutants by PCR；(C)-(H) The antibiotic susceptibility assays of ∆*lpaha1* and its site-directed mutants to OXY using the MBC plate method. The *lpaha1* mutants are numbered in the following order. 1. *A. hydrophila LP-2;* 2. *∆lpaha1; 3. ∆lpaha1+lpaha1;* 4. *∆lpaha1+vector;* 5*. ∆lpaha1+lpaha1-K57R;* 6. *∆lpaha1+lpaha1-K57Q;* 7*. ∆lpaha1+lpaha1-K187R;* 8*. ∆lpaha1+lpaha1-K187Q;* 9*. ∆lpaha1+lpaha1-K197R;* 10*. ∆lpaha1+lpaha1-K197Q.*

**Figure S3. The antibiotic susceptibility assay of *ΔcobB,* *ΔacuC* and *ΔAHA_0149* mutant strains against OXY.** (A) and (B) Growth curves of *A. hydrophila* mutants, which were treated with or without 0.5 μg/mL OXY, respectively.

**Figure S4. Confirmation of the aha1 gene complementary and Kace modifications sites strains by PCR.** DNA amplification from aha1 complementary, *aha1* carrying an empty vector and Kace modifications sites strains using universal primer pair F/R, respectively. (A): M. DNA Ladder (DL 2000);1. ∆*aha1*+paha1; 2. ∆*aha1*+vector；3. *aha1*-57R; 4. *aha1*-57Q; 5. ∆*aha1*+paha1-K99R; 6. ∆*aha1*+paha1-K99Q; 7. ∆*aha1*+paha1-K166R; 8. ∆*aha1*+paha1-K166Q; 9. ∆*aha1*+paha1-K175R; 10. ∆*aha1*+paha1-K175Q; 11. ∆*aha1*+paha1-K178R; 12. ∆*aha1*+paha1-K178Q; 13. ∆*aha1*+paha1-K187R; (B): M. DNA Ladder (DL 2000); 1. ∆*aha1*+paha1-K187Q; 2. ∆*aha1*+paha1-K197R; 3. ∆*aha1*+paha1-K197Q; 4. ∆*aha1*+paha1-K57R-K187Q-K197Q; 5. ∆*aha1*+paha1-K57Q-K187R-K197Q; 6. ∆*aha1*+paha1-K57Q-K187R-K197R; 7. ∆*aha1*+paha1-K57R-K187R-K197R; 8. ∆*aha1*+paha1-K57Q-K187Q-K197R; 9. ∆*aha1*+paha1-K57Q-K187Q-K197Q; 10. ∆*aha1*+paha1-K57R-K187R-K197Q; 11. ∆*aha1*+paha1-K57R-K187Q-K197R.

**Figure S5. Aha1, two deacetylases AcuC and CobB in *A. hydrophila* were purified and separated by SDS-PAGE.**

**Figure S6. MBCs results of Kace modification sites of AHA1 derivatives against seven types of a total 31 antibiotics.** The *aha1* derivatives were numbered in the following order. 1: WT; 2.Δ*aha1*; 3. Δ*aha1*+*paha1*; 4. Δ*aha1*+vector; 5. Δ*aha1*+*paha1-K57R*; 6. Δ*aha1*+*paha1-K57Q*; 7.Δ*aha1*+*paha1-K99R*; 8.Δ*aha1+paha1-K99Q*; 9.Δ*aha1*+*paha1-K166R*; 10.Δ*aha1*+*paha1-K166Q*; 11. Δ*aha1*+*paha1-K175R*; 12. Δ*aha1*+*paha1-K175Q*; 13.Δ*aha1*+*paha1-K178R*; 14.Δ*aha1*+*paha1-K178Q*; 15.Δ*aha1*+*paha1-K187R*; 16. Δ*aha1*+*paha1-K187Q*; 17. Δ*aha1*+*paha1-K197R*; 18.Δ*aha1*+*paha1-K197Q*; 19. Δ*aha1*+*paha1-K57R-K187Q-K197Q*;20.Δ*aha1*+*paha1-K57Q-K187R-K197Q*; 21.Δ*aha1*+*paha1-K57Q-K187R-K197R*; 22. Δ*aha1*+*paha1-K57R-K187R-K197R*; 23. Δ*aha1*+paha1-*K57Q-K187Q-K197R*; 24. Δ*aha1*+*paha1-K57Q-K187Q-K197Q*; 25.Δ*aha1*+*paha1-K57R-K187R-K197Q*; 26.Δ*aha1*+*paha1-K57R-K187Q-K197R*.

**Table S1. The primer pairs used in this study.**

**Table S2. Whole cell proteomics MS data.**

**Table S3. Kace peptide proteomics MS data.**

**Table S4. Keyword enrichment of altered proteins with up-down normalization score in whole-cell proteomics.**

**Table S5. Keyword enrichment of altered proteins with up-down normalization score in Kace proteomics.**

**Table S6. Motif analysis of Kac peptides in *A. hydrophila.***

**Table S7. differentially expressed protein KEGG pathways in the whole-cell proteomics.**
